# Antigen delivery by filamentous bacteriophage fd displaying an anti-DEC-205 single-chain variable fragment confers adjuvanticity by triggering a TLR9-mediated immune response

**DOI:** 10.15252/emmm.201404525

**Published:** 2015-04-17

**Authors:** Rossella Sartorius, Luciana D'Apice, Maria Trovato, Fausta Cuccaro, Valerio Costa, Maria Giovanna De Leo, Vincenzo Manuel Marzullo, Carmelo Biondo, Sabato D'Auria, Maria Antonietta De Matteis, Alfredo Ciccodicola, Piergiuseppe De Berardinis

**Affiliations:** 1 Institute of Protein Biochemistry, National Council of ResearchNaples, Italy; 2 Institute of Genetics and Biophysics A. Buzzati-Traverso, National Council of ResearchNaples, Italy; 3 Telethon Institute of Genetics and MedicinePozzuoli (NA), Italy; 4 Department of Pediatric, Gynecological, Microbiological and Biomedical Sciences, University of MessinaMessina, Italy; 5 Institute of Food Science, National Council of ResearchAvellino, Italy; 6 Department of Science and Technology, University Parthenope of NaplesNaples, Italy

**Keywords:** antigen delivery, DEC-205, dendritic cells, filamentous bacteriophage, TLR9

## Abstract

Filamentous bacteriophage fd particles delivering antigenic determinants via DEC-205 (fdsc-αDEC) represent a powerful delivery system that induces CD8^+^ T-cell responses even when administered in the absence of adjuvants or maturation stimuli for dendritic cells. In order to investigate the mechanisms of this activity, RNA-Sequencing of fd-pulsed dendritic cells was performed. A significant differential expression of genes involved in innate immunity, co-stimulation and cytokine production was observed. In agreement with these findings, we demonstrate that induction of proinflammatory cytokines and type I interferon by fdsc-αDEC was MYD88 mediated and TLR9 dependent. We also found that fdsc-αDEC is delivered into LAMP-1-positive compartments and co-localizes with TLR9. Thus, phage particles containing a single-strand DNA genome rich in CpG motifs delivered via DEC-205 are able to intercept and trigger the active TLR9 innate immune receptor into late endosome/lysosomes and to enhance the immunogenicity of the displayed antigenic determinants. These findings make fd bacteriophage a valuable tool for immunization without administering exogenous adjuvants.

## Introduction

DEC-205 has been classified as a type I C-type lectin receptor on the basis of sequence alignments (East & Isacke, 2002). It has a N-terminal cysteine-rich domain, a fibronectin type II domain and 10 C-type lectin domains and is mainly expressed on dendritic cells (DCs) (Jiang *et al*, 1995; Swiggard *et al*, 1995). It is not known which carbohydrate is recognized by DEC-205, and it has been proposed that it may function as a promiscuous antigen receptor (Jiang *et al*, 1995; Mahnke *et al*, 2000). More recently, it has been reported that DEC-205 is a cell surface receptor for CpG oligonucleotides (Lahoud *et al*, 2012). DEC-205 has an internalization sequence in its cytoplasmic tail and thus has endocytic capacity. It recycles through late endosomal/lysosomal compartments (Mahnke *et al*, 2000) facilitating the presentation of antigens targeted to DCs with antigen-bearing monoclonal antibodies in the presence of strong adjuvants such as CpG (Mahnke *et al*, 2005), polyinosinic-polycytidylic acid (Poly I:C) (Trumpfheller *et al*, 2008) or anti-CD40 antibody (Bonifaz *et al*, 2004). Based on these properties, the use of antigen-conjugated anti-DEC monoclonal antibody (mAb) together with adjuvant administration has been proposed for targeted vaccination strategies (Van Broekhoven *et al*, 2004). However, signals for DC activation are not induced by anti-DEC-205 antibodies, and their use in antigen delivery in the absence of exogenously added adjuvants induces tolerance (Hawiger *et al*, 2001; Bonifaz *et al*, 2002; Mahnke *et al*, 2003).

Given this scenario, we previously described the construction of phage particles able to deliver antigen determinants via DEC-205. We also reported that the administration of bacteriophage virions in the absence of adjuvants conferred immunogenicity to the displayed epitopes (Sartorius *et al*, 2011).

The system of antigen delivery by bacteriophage is based on modification of the phage display technology. The filamentous bacteriophage fd is well understood at both the structural and genetic level (Banner *et al*, 1981; Hunter *et al*, 1987; Straus *et al*, 2008). Bacteriophages only infect and multiply with their specific host, and currently, the therapeutic use of bacteriophages is back on the agenda as bacterial resistance to antibiotics becomes widespread (Reardon, 2014). It should be mentioned that the use of the bacteriophage ΦX174 to assess specific antibody responses in patients with immunodeficiencies has been reported for many years, and is considered a safe, well-tolerated and clinically useful method (Smith *et al*, 2014). In theory, the administration of filamentous bacteriophage fd in humans should be also considered safe, even if no human tests with this type of recombinant bacteriophage as a delivery system have been performed so far.

Overall, immunization with filamentous phages displaying antigenic peptides has been described as being able to elicit a protective immune response in animal models of disease (van Houten *et al*, 2006, 2010).

Filamentous bacteriophages are taken up and processed by the major histocompatibility complex (MHC) class I and II pathways (Gaubin *et al*, 2003) and are also able to induce an immune response mediated by cytotoxic T lymphocytes (De Berardinis *et al*, 1999, 2000; D'Apice *et al*, 2007; Sartorius *et al*, 2008). Importantly, we showed that phage particles displaying single-chain variable fragment (scFv) of an anti-DEC-205 monoclonal antibody were targeted to DCs and were able to induce maturation, even in the absence of maturation stimuli (Sartorius *et al*, 2011). Thus, the fd antigen delivery system combines the safety and capability to trigger a strong cellular antigen-specific immune response even in the absence of adjuvants.

In this work, taking advantage of next-generation sequencing (NGS) analysis, we dissected the transcriptome changes occurring in dendritic cells after exposure to fdsc-αDEC bacteriophage, and by observing the intracellular fate of fd virions targeting DEC-205, we provide insights into the immunogenicity of fd particles delivered to dendritic cells in the absence of exogenously added maturation stimuli.

## Results

### Immunogenicity of the ovalbumin (OVA) peptide OVA_257-264_ delivered via DEC-205

**Figure 1:**
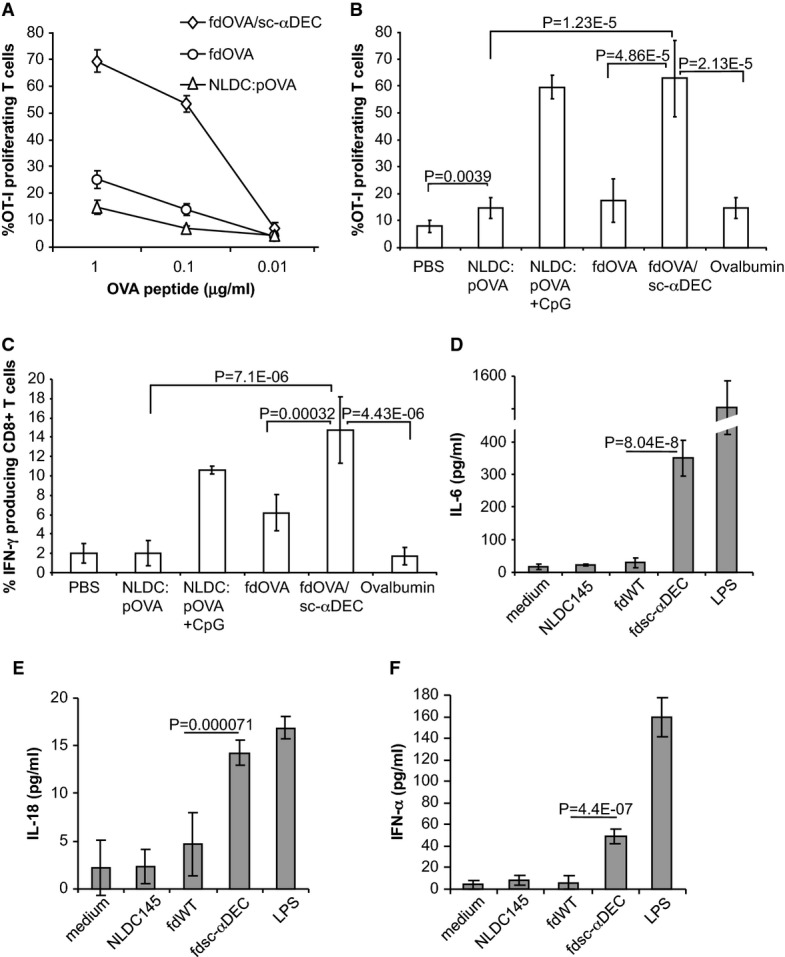
OVA_257-264_ -specific T-cell response and cytokine production

We analyzed the ability of immature bone marrow-derived dendritic cells (BMDCs) loaded with lipopolysaccharide (LPS)-free fd particles displaying the peptide OVA_257-264_ (SIINFEKL) (fdOVA) or fd particles co-displaying the peptide and the anti-DEC-205 scFv (fdOVA/sc-αDEC) to stimulate antigen-specific CD8^+^ T cells. For comparative analysis, BMDCs were loaded with the same molar amount of OVA_257-264_ peptide delivered by the anti-DEC-205 mAb (NLDC:pOVA). Carboxyfluorescein succinimidyl ester (CFSE)-labeled OVA_257-264_-specific TCR transgenic T cells (CD8^+^ MHC class I-restricted OT-I T cells) were used as responder cells in a proliferation assay. Fig1A shows the proliferative activity of CD8^+^ OT-I T cells after three days of co-culture with DCs that had been prepulsed with the antigenic preparations mentioned above. A higher proliferative response was induced by fdOVA/sc-αDEC particles compared to fdOVA (non-targeted OVA_257-264_ bacteriophage particles) and to NLDC:pOVA_257-264_ (OVA_257-264_ peptide delivered by anti-DEC mAb).

A Specific CD8^+^ T-cell (OT-I) proliferation in response to immature BMDCs incubated with fdOVA, fdOVA/sc-αDEC or OVA_257-264_ peptide chemically coupled to anti-DEC-205 antibody (NLDC:pOVA). Percentage of CFSE-labeled proliferating T cells was evaluated on CD8^+^-gated cells after 72 h of DC:T co-culture. The mean ±SD of three independent experiments is reported.

B Proliferation of adoptively transferred OT-I T cells in C57BL/6 mice (*n* = 3/group) immunized with vehicle (PBS), NLDC:pOVA, NLDC:pOVA plus CpG, fdOVA, fdOVA/sc-αDEC or LPS-free ovalbumin protein. Percentage of CFSE-labeled proliferating OT-I T cells was evaluated after three days on Vα2^+^ CD8^+^-gated cells. The mean ± SD of two independent experiments is reported. Comparative analyses were performed using Student's *t*-test for unpaired samples.

C Percentage of IFN-γ-producing CD8^+^ splenocytes isolated from mice immunized as in (B). The mean ± SD of two independent experiments is reported. Comparative analyses were performed using Student's *t*-test for unpaired samples.

D–F IL-6 (D), IL-18 (E) and IFN-α (F) release in supernatants of BMDCs obtained from C57BL/6 mice. Supernatants of BMDCs incubated with NLDC145 antibody, wild-type phage particles (fdWT) or scFv αDEC-205 phage particles (fdsc-αDEC) for 20 h were analyzed by ELISA for cytokine production. Unstimulated culture (medium) and LPS-treated culture were, respectively, used as negative and positive controls. Bars represent mean values ± SD. Cumulative results are shown of three independent experiments assayed in duplicate. Comparative analyses were performed using Student's *t*-test for unpaired samples.

We also performed adoptive transfer experiments to compare the immune response to the OVA_257-264_ peptide delivered to DCs by bacteriophages or by the anti-DEC-205 mAb. C57BL/6 mice, transferred with CFSE-labeled antigen-specific OT-I T cells, were injected with NLDC:pOVA or fd particles expressing the OVA_257-264_ determinant (fdOVA or fdOVA/sc-αDEC) containing 1.6 μg of OVA_257-264_. As a control, mice were also injected with 50 μg of soluble LPS-free ovalbumin protein or with NLDC:pOVA conjugate plus CpG as adjuvant. After 3 days, OVA_257-264_-specific OT-I proliferation and IFN-γ production were evaluated. A strong proliferative response and IFN-γ production were found using fdOVA/sc-αDEC bacteriophage particles. The fdOVA particles (not displaying anti-DEC-205 scFv) or the NLDC:pOVA (OVA_257-264_ peptide delivered in the same molar amount by NLDC145 mAb) induced a significantly lower antigen-specific OT-I CD8^+^ T-cell proliferative response (Fig1B) and no or weaker IFN-γ production (Fig1C).The proliferative activity induced by the OVA_257-264_ antigenic determinant delivered by anti-DEC-205 is low in comparison with a previous report describing the proliferation of OT-I T cells stimulated by an anti-DEC-205 antibody carrying ovalbumin protein (Bonifaz *et al*, 2004), while in agreement with this report (which showed lack of IFN-γ production using ovalbumin conjugated to an anti-DEC-205 antibody), we did not observe production of IFN-γ by OT-I T cells stimulated with anti-DEC antibody delivering the OVA_257-264_ peptide (NLDC:pOVA, Fig1B and C). When NLDC:pOVA conjugate was administered in the presence of the adjuvant CpG, a higher proliferative response as well as IFN-γ production by OT-I T cells was observed (Fig1B and C). These data indicate that, in an experimental setting characterized by the absence of exogenously added adjuvants, the fd bacteriophage DEC-205-targeted particles confer strong immunogenicity to the displayed antigenic determinant.

We also observed that bacteriophage particles delivered via anti-DEC-205 scFv are internalized by dendritic cells more efficiently than non-targeted virions (Supplementary Fig S1). More than 60% of dendritic cells were fluorescein isothiocyanate (FITC) positive after incubation of BMDCs with FITC-conjugated bacteriophages expressing scFv anti-DEC-205 (fdsc-αDEC) for 30 min; this level of internalization is comparable to the value obtained for anti-DEC-205 monoclonal antibody internalization. By contrast, non-targeted fd wild-type bacteriophages (fdWT) were internalized less efficiently since only the 30% of DCs were FITC positive under the same conditions.

Importantly, BMDCs pulsed with fdsc-αDEC phage particles were able to produce IL-6 (Fig1D), IL-18 (Fig1E) and IFN-α (Fig1F) while BMDCs pulsed with fdWT particles did not produce these cytokines (Fig1D–F). In accordance with previous findings (Bonifaz *et al*, 2002), NLDC145 mAb in the absence of simultaneous administration of other stimuli did not elicit cytokine production (Fig1D–F).

### Transcriptome analysis by RNA-Sequencing

In order to understand the different activity shown by bacteriophage particles delivered via DEC-205, we analyzed the transcriptional profiles of DCs exposed to fdWT or fdsc-αDEC phage particles using RNA-Sequencing (RNA-Seq). Two technical replicates for each condition (DCs treated with fdWT and fdsc-αDEC) were performed. Approximately 55 million reads (95% of them uniquely mapped on the reference genome) were produced for each replicate. Expression values from the biological replicates obtained for fdWT and fdsc-αDEC, computed as FPKM (fragments per kilobase of transcript and million of mapped reads), were highly correlated (Supplementary Fig S2).

**Figure 2:**
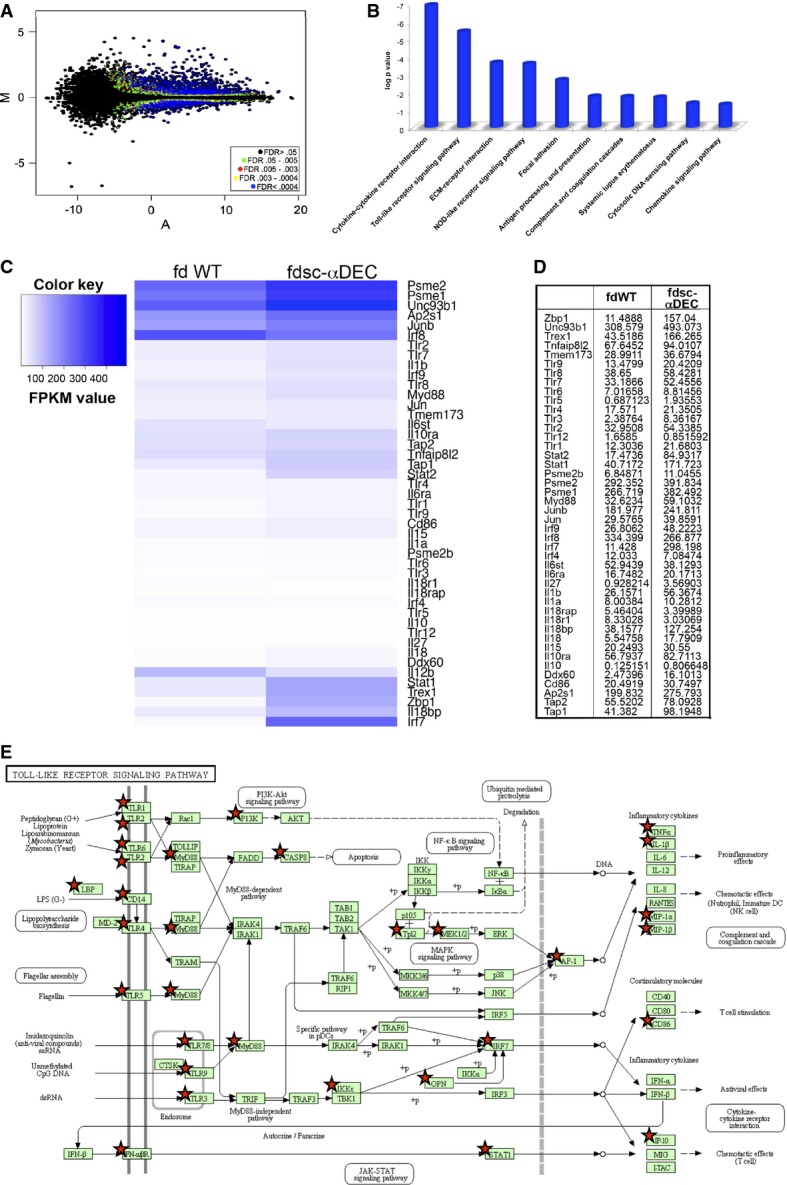
RNA-Seq of bacteriophage-pulsed dendritic cells
A Standard MA plot (M, log ratios and A, mean average) of the FPKM (fragments per kilobase of transcript and million of mapped reads) of fdWT vs fdsc-αDEC-treated dendritic cells, for each RefSeq gene. The *y*-axis represents the log fold change (M) and the *x*-axis the average log intensity (A) for each gene (dot). Colored dots indicate differentially expressed genes. As indicated in the inset, blue dots constitute the ‘stringent set’ of differentially expressed genes (FDR <0.0004).

Using RNA-Sequencing, we simultaneously analyzed gene expression of all genes annotated in the mouse genome. Using a threshold for gene expression levels (FPKM = 1), about 10,000 genes were expressed at significant levels in both conditions. We compared gene expression levels between the two conditions and detected approximately 4,000 genes that were differentially expressed in DCs after exposure to fdsc-αDEC compared to cells treated with fd wild-type (Fig2A). Furthermore, to understand—in an unbiased way—whether the differentially expressed genes are significantly related to specific gene ontologies and to specific cellular pathways, we analyzed (using the Database for Annotation, Visualization and Integrated Discovery, DAVID) a stringent set of differentially expressed genes (false discovery rate (FDR) < 0.0004; see Fig2A, blue dots). The most enriched biological pathways (KEGG database) are shown in Fig2B. Interestingly, we observed that the exposure of DCs to fdsc-αDEC significantly up-regulated many genes that are involved in inflammatory pathways linked to innate immunity. Most relevant pathways were the cytokine–cytokine receptor and chemokine signaling pathways, the ‘Toll-like receptors’ (TLRs) and the ‘NOD-like receptor’ signaling pathways, the ‘DNA sensing’ and the ‘antigen processing/presentation’ (Fig2B).

B Bar graph showing the most significant gene pathways enriched with up-regulated genes of the ‘stringent set’ in the fdWT vs fdsc-αDEC comparison.

C, D Heatmap (C) and table (D) of FPKM values for selected genes in fdWT and fdsc-αDEC-pulsed DCs.

E Schematic representation of the ‘Toll-like receptor signaling pathway’ (KEGG pathway 04620), significantly altered in fdsc-αDEC-treated cells. Red stars indicate the genes of the ‘stringent set’ that are significantly (FDR < 0.0004) up-regulated in fdsc-αDEC compared to fdWT.

The heatmap in Fig2C shows a comparison of normalized expression values of selected genes between fdWT and fdsc-αDEC-pulsed dendritic cells, with the FPKM values reported in Fig2D. DCs treated with fdsc-αDEC mainly up-regulate genes encoding immune-related cytokines and chemokines, including *Cxcl10*, *Il1b*, *Il18* and *Il15*, and the gene encoding the co-stimulatory molecule CD86. Moreover, many interferon signaling genes such as *Ifnar2*, *Stat1*, *Stat2* and *Irf7* are differentially expressed, indicating the development of type 1 immuno-stimulatory dendritic cells (DC1).

RNA-Seq data also revealed the up-regulation of the expression of the two MHC-linked genes *Tap1* and *Tap2* that are required for the antigen-processing and presentation pathway of intracellular antigens to T cells, and of *Psme1/2* genes encoding the immune-proteasome-associated complex PA28 subunits alpha and beta. PA28β expression is low in immature DCs and strongly increases in mature DCs (Ossendorp *et al*, 2005), and more recently, it has been reported that the immunoproteasome has a major role in regulating gene transcription in maturing DCs (de Verteuil *et al*, 2014).

Our transcriptome data also show that, compared to fd wild-type, fdsc-αDEC induces the expression of genes encoding DNA sensor proteins such as STING (*Tmem173*), DAI (*Zbp1*) and TREX1 (*Trex1*) in dendritic cells. TREX1 has been described as being up-regulated by TLR ligands in murine conventional dendritic cells (Xu *et al*, 2014), and, in this context, we detected a significant up-regulation of TLR transcripts, including TLR9 which also senses DNA (Supplementary Fig S3). Moreover, we observed a significant up-regulation of transcripts encoding proteins linked to the TLR pathway (summarized in Fig2E), such as the MYD88 adaptor molecule (Supplementary Fig S3), and proteins such as UNC93B1 involved in the trafficking of endosomal TLRs (TLR3, TLR7 and TLR9) (Supplementary Fig S3). These findings prompted us to investigate the role of TLRs after DC exposure to fdsc-αDEC.

### Role of MYD88 and TLR9 in DCs targeted by phage particles displaying anti-DEC-205 scFv

**Figure 3:**
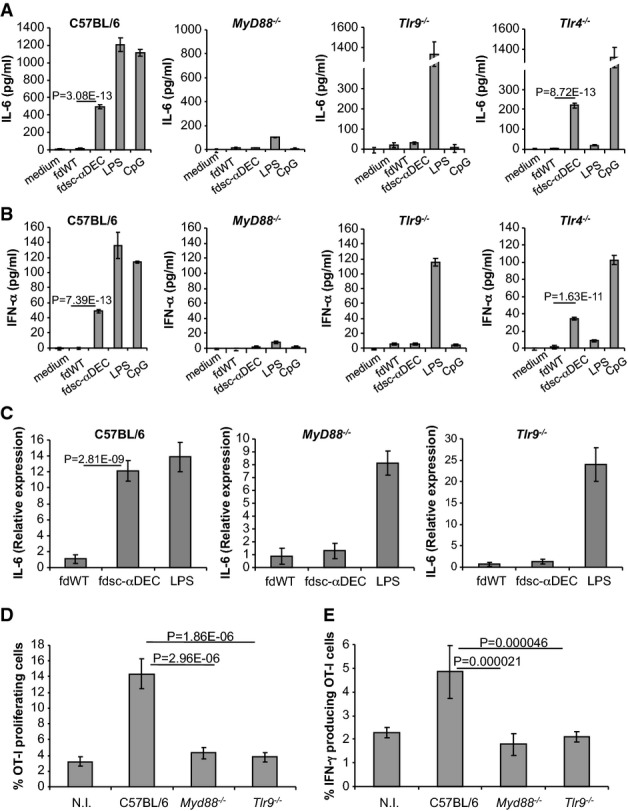
fdsc-αDEC induces IL-6 and IFN-α production mediated by MYD88 and TLR9
IL-6 was evaluated by ELISA in supernatants of BMDCs obtained from C57BL6, MYD88, TLR9 or TLR4 KO mice and incubated for 20 h with wild-type or fdsc-αDEC phage particles. LPS or CpG-ODN were used as controls. IL-6 release from DCs derived from *MyD88*^*−/−*^ mice was totally abolished and dramatically reduced in DCs derived from *Tlr9*^*−/−*^ mice, but not affected in *Tlr4*^*−/−*^ DCs. Bars represent mean values ± SD. Cumulative results are shown of three independent experiments assayed in duplicate. Comparative analyses were performed using Student's *t*-test for unpaired samples.

The involvement of the TLR pathway in the activity induced by fdsc-αDEC particles after their internalization and/or degradation in the endolysosomal compartments was initially investigated by analyzing the production of the pro-inflammatory cytokine IL-6 in *MyD88*^*−/−*^ DCs pulsed with LPS-free phage virions. As illustrated in Fig3A, the production of IL-6 by fdsc-αDEC was totally abolished in *MyD88*^*−/−*^ DCs compared to wild-type BMDCs, confirming the involvement of the TLR pathway. Since the filamentous phage particles contain a single-strand (ss) DNA rich in unmethylated CpG sequences, and since TLR9 recognizes unmethylated CpG motifs of bacterial and viral ssDNA, we next specifically investigated the role of TLR9 in the induction of cytokine production after phage uptake.

IFN-α release evaluated by ELISA in supernatants of BMDCs obtained from C57BL/6 or KO mice and incubated for 20 h with wild-type or fdsc-αDEC phage particles. LPS or CpG-ODN were used as controls. *MyD88*^*−/−*^ and *Tlr9*^*−/−*^ but not *Tlr4*^*−/−*^ BMDCs were unable to produce IFN-α after fdsc-αDEC stimulation. Bars represent mean values ± SD. Cumulative results are shown of three independent experiments assayed in duplicate. Comparative analyses were performed using Student's *t-*test for unpaired samples.

IL-6 mRNA expression by DC cells. C57BL/6, *MyD88*^*−/−*^ and *Tlr9*^*−/−*^ mice were inoculated intraperitoneally with fdWT or fdsc-αDEC bacteriophages or, as a control, with LPS. Mice were sacrificed 2 h later, and purified spleen dendritic cells were analyzed for IL-6 mRNA levels by quantitative real-time PCR. Bars represent the mean fold increase ± SD. The experiments were performed three times (*n* = 2 per group). Comparative analyses were performed using Student's *t-*test for unpaired samples.

Proliferation of OT-I CD8^+^ T cells after 72 h of co-culture with 1 × 10^4^ DCs isolated from C57BL/6 mice or from *MyD88*^*−/−*^ or *Tlr9*^*−/−*^ transgenic mice previously injected with fdOVA/sc-αDEC bacteriophage particles. The panel shows the percentage of divided, CFSE-low OT-I CD8^+^ T cells. As a control, the proliferation of OT-I CD8^+^ T cells co-cultured with DCs isolated from non-immunized (NI) C57BL/6 mice is reported. The mean ± SD of two independent experiments with *n* = 3 per group is reported. Comparative analyses were performed using Student's *t*-test for unpaired samples.

IFN-γ production by OT-I CD8^+^ T cells co-cultured for 12 h in the presence of 3.3 × 10^3^ DCs isolated from C57BL/6 mice or MyD88^−/−^ and Tlr9^−/−^ transgenic mice injected as in (D). Data information: The mean ± SD of two independent experiments with *n* = 3 per group is reported. Comparative analyses were performed using Student's *t-*test for unpaired samples.

To this end, we measured IL-6 production in the supernatants of BMDCs isolated from *Tlr9*^*−/−*^ mice that had been co-cultured with fdWT or fdsc-αDEC bacteriophage particles. We found that IL-6 release is severely impaired using fdsc-αDEC bacteriophages in DCs isolated from mice lacking TLR9 expression but not in DCs lacking TLR4, used as a control (Fig3A). Interestingly, IFN-α release also appears to be linked to TLR9 signaling, since both *MyD88*^*−/−*^ and *Tlr9*^*−/−*^ DCs, but not *Tlr4*^*−/−*^ DCs, are unable to produce IFN-α when pulsed with fdsc-αDEC bacteriophages (Fig3B).

Furthermore, we also assessed inflammatory cytokine production in DCs isolated from immunized mice. We injected C57BL/6 mice with LPS-free fdWT or fdsc-αDEC bacteriophages. Two hours later, DCs were isolated from the spleen of immunized mice by magnetic separation, total RNA was extracted, and the expression level of IL-6 mRNA was assessed using quantitative real-time (RT) PCR. The relative gene expression was calculated using the 2^−ΔΔCt^ method (Livak & Schmittgen, 2001), with PBS-treated mice as calibrator and β-actin as a housekeeping gene. As shown in Fig3C, delivering fd bacteriophage via DEC-205 scFv resulted in a strong up-regulation of IL-6 mRNA expression (up to 12-fold), while DCs isolated from mice treated with fdWT bacteriophages showed no increase.

Moreover, we measured IL-6 mRNA levels in splenic DCs isolated from *MyD88*^*−/−*^ or *Tlr9*^*−/−*^ mice 2 h after fdWT or fdsc-αDEC injection. As reported in Fig3C, negligible levels of IL-6 were produced in either *MyD88*^−/−^ or *Tlr9*^*−/−*^ DCs after wild-type or fdsc-αDEC bacteriophage injection, indicating that dendritic cells targeted via anti-DEC-205 bacteriophages *in vivo*, analogously to *in vitro* treated BMDCs, are able to produce proinflammatory cytokines via TLR9/MYD88 signaling.

Finally, splenic DCs were purified by magnetic separation 18 h after the administration of the phage particles to C57BL/6, *MyD88*^*−/−*^ and *Tlr9*^*−/−*^ mice and co-cultivated with CFSE-labeled OVA_257-264_-specific OT-I transgenic T cells. DCs isolated from C57BL/6 mice and targeted *in vivo* with fdOVA/sc-αDEC induced significant OT-I proliferation (Fig3D) and IFN-γ production (Fig3E), whereas DCs isolated from *MyD88*^*−/−*^ and *Tlr9*^*−/−*^ mice promoted neither CD8^+^ OT-I proliferation nor IFN-γ release.

These results demonstrate that the ability of fdOVA/sc-αDEC phage particles to induce cytokines production and to confer higher immunogenicity to the displayed antigenic determinants is dependent on activation of the TLR9/MYD88 pathway.

### Subcellular localization of fd particles

**Figure 4:**
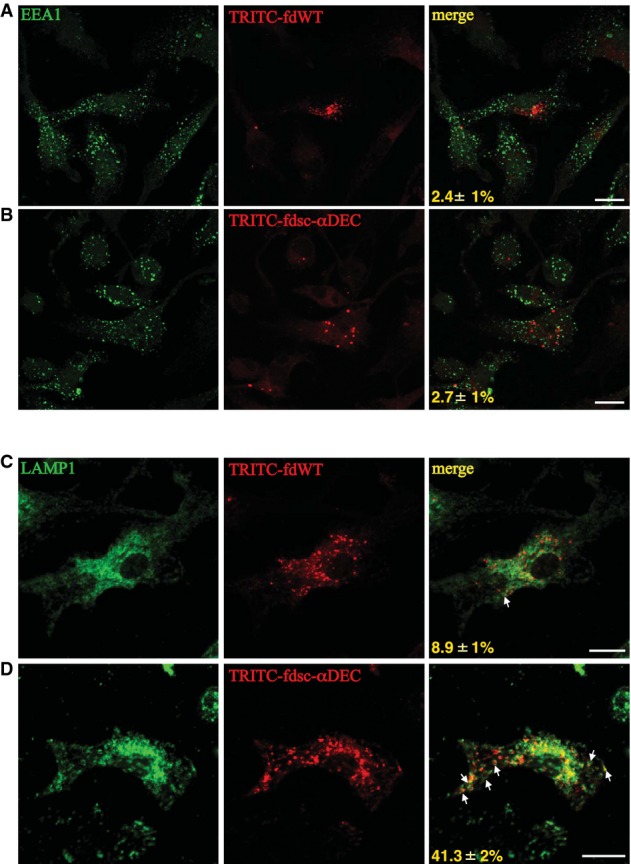
Confocal microscopy analysis of BMDCs after phage uptake
A–D BMDCs were incubated with TRITC-conjugated fdWT (A, C) or fdsc-αDEC bacteriophages (B, D) for 6 h. Cells were then fixed, permeabilized, immunostained with anti-EEA-1 (A, B) or anti-LAMP-1 (C, D) (green) antibody and analyzed by confocal microscopy. Representative images are shown. Numbers at the bottom of the right panels indicate the percentage of EEA-1 (A, B) or LAMP-1 (C, D) bacteriophage co-localization (mean values ± SD, *N* = 2, *n* = 100). Scale bars, 10 mm. White arrows indicate LAMP-1-positive structures that also are positive or contain fdsc-αDEC or fdWT bacteriophages.

In order to understand how and where fdsc-αDEC intercepts TLR9, we analyzed the intracellular fate of fdsc-αDEC and of wild-type bacteriophages by dissecting the intracellular trafficking route of fd particles (delivered or not via DEC-205) in BMDCs. BMDCs incubated with tetramethylrhodamine (TRITC)-conjugated fdWT or fdsc-αDEC for 6 h were analyzed by confocal microscopy, and their endosomal compartments were mapped using early endosomal (EEA-1) or late endosomal/lysosomal (LAMP-1) markers. We observed low co-localization of fd bacteriophages particles, either wild-type or expressing anti-DEC-205, in EEA-1-positive early endosomes (< 3% of EEA-1-positive structures contain fd wild-type (Fig4A) or fdsc-αDEC phage particles (Fig4B)). By contrast, we found that fdsc-αDEC bacteriophages, but not fd wild-type, localized in the LAMP1-positive late endosomal/lysosomal compartments. As shown in Fig4C and D, the display of anti-DEC-205 scFv on the phage surface markedly increases the localization of bacteriophages into late endolysosomal compartments. Co-localization analysis showed that 41.3% of LAMP-1-positive structures also contain fdsc-αDEC phage particles (Fig4D), while fd wild-type seems to localize with only 8.9% of the LAMP-1-positive endolysosomes (Fig4C).

These data indicate that fd particles displaying the anti-DEC-205 scFv behave like the anti-DEC-205 antibody that has been described to deliver antigens to late endosomal/lysosomal compartments (Platt *et al*, 2010).

**Figure 5:**
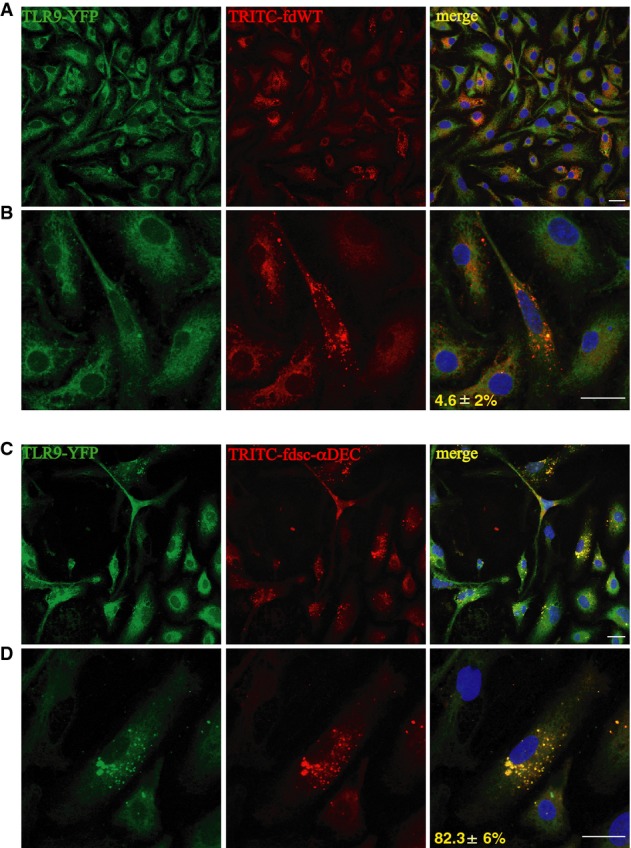
fdsc-αDEC localizes with TLR9 in DCs
A–D BMDCs from C57BL/6 mice were stably transduced with YFP-tagged TLR9. Cells grown on coverslips were then incubated with TRITC-conjugated wild-type (A, B) or fdsc-αDEC (C, D) bacteriophages for 6 h. Cells were then washed, fixed and analyzed by confocal microscopy. Nuclei were counterstained with Hoechst. (B, D) are enlargements of a single cell. Representative images are shown. Numbers at bottom of right panels indicate the percentage of TLR9 bacteriophage co-localization (mean values ± SD, *N* = 2, *n* = 100). Scale bars, 10 mm.

To verify the co-localization of phage particles internalized via the DEC-205 receptor with TLR9 in dendritic cells, we expressed recombinant TLR9 C-terminally fused to yellow fluorescent protein (YFP) in BMDCs. Using TRITC-conjugated bacteriophages and confocal microscopy, we were able to assess the co-localization of phage particles internalized by DCs with the TLR9-YFP. We found that fdsc-αDEC particles, which are able to activate DCs, induce a high number of TLR9-positive structures compared to cells incubated with fd wild-type particles (Fig5A and C, left panels). Moreover, the majority (82.3%) of the fdsc-αDEC virions co-localized with TLR9 (Fig5C and D) compared to only 4.6% co-localization of the fd wild-type bacteriophages (Fig5A and B).

These data of co-localization indirectly suggest that phage particles, containing a single-strand DNA genome rich in CpG motifs, when delivered via DEC-205, are able to intercept and trigger the active TLR9 innate immune receptor into the late endolysosomal compartments, and thus to enhance the immunogenicity of the displayed antigenic determinants.

## Discussion

The use of *ex vivo* generated DCs pulsed with tumor antigens represents a promising immunotherapeutic option which is the object of ongoing clinical trials (Palucka & Banchereau, 2012). However, this type of vaccination is expensive and not easy to perform. A more promising approach could be the direct *in vivo* targeting of dendritic cells by coupling antigens to antibodies able to recognize receptors that are specifically expressed by DCs. In this context, it has been described that antigen-conjugated antibodies targeting C-type lectin receptors such as DEC-205 that are expressed by DCs efficiently induce antigen-specific T-cell responses (Bonifaz *et al*, 2004). However, in the case of DEC-205, this delivery is efficient only if associated with the administration of maturation stimuli for DCs, such as anti-CD40 or Poly I:C, and it is known that administration of these adjuvants may have side effects (Cornell *et al*, 1976; Levine & Levy, 1978; Cairing *et al*, 2005). To bypass this constraint, we reported that antigen-displaying filamentous bacteriophages targeting DEC-205 induce strong and protective antigen-specific T-cell responses (Sartorius *et al*, 2011). These data indicate that vaccination with phage targeted to DEC-205 does not need addition of exogenous adjuvants and that scFv anti-DEC-205 phage particles have per se adjuvant properties, being able to induce the production of pro-inflammatory cytokines and co-stimulatory molecules in DCs and to induce DC maturation (Sartorius *et al*, 2011). It is known that antigen presentation and co-stimulation provided by DCs to T cells as well as cytokines secreted by DCs determine T-cell activation. The conversion of DCs from tolerogenic to immunogenic (e.g., during infectious diseases) is thought to depend on their capacity to sense pathogen components and to produce cytokines. It is also known that cytokine gene transcription in DCs depends on the activation of transcription factors such NF-kB, Ap-1 or IRF induced by pattern recognition receptor molecules (PRR) such as the Toll-like receptors and C-type lectin receptors. However, literature data indicate that the DEC-205 type I C-type lectin receptor is not able to signal and activate transcription factors, while it is efficient in favoring the internalization of target antigens (Mahnke *et al*, 2000; Cohn *et al*, 2013). In contrast, our data show that phage particles targeting the DEC-205 receptor are able to trigger signaling in DCs. In order to investigate at the transcriptional level the mechanism of action of fdsc-αDEC particles, we used RNA-Sequencing technology on DCs after exposure to fdsc-αDEC or to wild-type fd. This analysis revealed that phages delivered via DEC-205 are able to significantly induce the expression of relevant gene pathways involved in DC activation and in the induction of efficient Th1-mediated immune responses. Here, we also describe that the activity of fdsc-αDEC particles is dependent on the recruitment of the adaptor molecule MYD88. MYD88 is essential for the downstream signaling of various TLRs such as TLR9 (Häcker *et al*, 2000), and our data also demonstrate the role of TLR9 in the functional activity of DEC-205-targeted phage virions. TLR9 senses unmethylated single-stranded DNA with CpG motifs derived from bacteria and viruses (Hemmi *et al*, 2000; Mason *et al*, 2005), and phage virions contain a single-stranded genome rich in CpG motifs.

Interestingly, the RNA-Seq data indicate that phage particles addressed to late endosomes by DEC-205 are also able to up-regulate the transcription factor IRF7, which is upstream of IFN-α production, while wild-type phage (not able to access late endosomes) does not induce IRF7 up-regulation nor the production of IFN-α by DCs. Type I IFN is generally known to be produced by plasmacytoid DCs (pDCs) and the retention of signaling complexes in early endosome in pDCs rather than in late endosomes or lysosomes in other cell subsets have been correlated with IRF7 recruitment (Honda *et al*, 2005; Blasius & Beutler, 2010). It should be emphasized that mouse pDCs do not express DEC-205 (Benitez-Ribas *et al*, 2011), and we have previously described the detection of IFN-α in the sera of mice immunized with fdsc-αDEC (Sartorius *et al*, 2011). Here, we provide evidence that phage particles targeted to late endosomes in BMDCs via DEC-205 are indeed able to up-regulate IRF7 and induce type I IFN production through the involvement of TLR9 and MYD88.

The majority of TLR9 is localized in the endoplasmic reticulum of DC cells before stimulation and is rapidly recruited to late endosomal/lysosomal LAMP-1-positive compartments upon activation by CpG DNA (Leifer *et al*, 2004). Only a small fraction of TLR9 constitutively moves through the cell and reaches the lysosomal compartment (Kim *et al*, 2008; Chockalingam *et al*, 2009) to provide a pool of TLR9 immediately ready to respond to endocytosed CpG DNA. This small fraction provides an initial signal, but to provide the optimal signal strength, additional TLR9 translocation from the ER to endolysosomes is required. Accordingly, we found that fdsc-αDEC, which is able to activate dendritic cells and is found in LAMP-1-positive compartments after DC internalization, is able to localize in the same compartments where TLR9 is found, while fd wild-type particles do not show a high level of co-localization with TLR9.

In order to become active, the ectodomain of TLR9 is cleaved in the endolysosome such that no full-length TLR9 is detectable in the endolysosomal compartment (Latz *et al*, 2004; Park *et al*, 2008). Importantly, only the cleaved form of TLR9 recruits MYD88 on activation (Ewald *et al*, 2008). It has been described that TLR9 signals may also lead to the activation of type I IFN and this activity requires TLR9 trafficking to a specialized compartment named the lysosome-related organelle (Sasai *et al*, 2010).

Thus, we hypothesize that phage ssDNA, targeted to DCs by DEC-205, interacts with TLR9 in late endosomes to induce the production of pro-inflammatory cytokines, and it is also delivered to lysosomal-related organelles where it signals IRF7 to induce the production of type I IFN.

The interaction between active TLR9 and phage DNA delivered via DEC-205 in endolysosomes is also supported by the fact that wild-type phage particles, which enter endolysosomal compartments only in limited amounts, are not able to elicit the production of pro-inflammatory cytokines.

Moreover, the transcriptome analysis described herein shows that DC exposure to fdsc-αDEC, but not to the wild-type bacteriophages, is able to induce up-regulation of *Unc93b1*, a gene encoding a multi-pass transmembrane protein required for TLR9 trafficking from the ER to endolysosomes. UNC93B1 remains associated with TLR9 even after Golgi processing, and the mechanism by which UNC93B1 controls the traffic of a functional TLR9 to endolysosomes has been clarified recently (Lee *et al*, 2013). In particular, these authors showed that sorting of endosomal TLR9 by UNC93B1 requires the recruitment of the adaptor protein complex (AP-2). Notably, the genes encoding two subunits of this complex (*Ap2s1* and *Ap2a2*) are up-regulated in BMDCs pulsed by fdsc-αDEC. The TLRs that recognize nucleic acids are compartmentalized to avoid unwanted activation, since discrimination between self and microbial nucleic acids is not achieved solely through recognition of distinct features but relies on differential delivery of these potential ligands to TLRs (de Jong *et al*, 2010; Chockalingam *et al*, 2012). The interaction of TLR9 with unmethylated phage ssDNA in endolysosomes may benefit from the presence of a proteinaceous coat shielding the phage ssDNA from the degradative activity of intracellular DNase. However, we can also hypothesize that phage ssDNA can escape from endosomal compartments and leak into the cytoplasm, thus activating DNA sensing molecules in the cytoplasm of fdsc-αDEC-pulsed DCs that lead to IRF7 signaling and type I IFN production.

In this context, RNA-Seq data show the up-regulation of the DNA sensing pathway and related molecules such as STING, DAI and TREX1. However, since the production of IFN-α after fdsc-αDEC stimulation is not detectable in MYD88-negative DCs, it is likely that the induction of the IRF7 transcription factor is mediated by TLR9, rather than by the above-mentioned DNA sensing molecules.

Overall, we have shown that fdsc-αDEC phage particles traffic to endolysosomal compartments and co-localize with TLR9. In contrast, phage particles not displaying DEC-205 do not reach endolysosomal compartments and do not co-localize with TLR9. Phage delivered to the endolysosomes via DEC-205 is able to induce up-regulation of different gene expression pathways and to trigger signals through TLR9 and MYD88. As a consequence, the delivery of phage single-strand DNA in endolysosomes by fdsc-αDEC bacteriophage particles induces cytokine production and DC maturation and, as we described previously (Sartorius *et al*, 2011), strong and protective immune responses toward the displayed antigens.

Future work using fd bacteriophages displaying anti-human DEC-205 scFv will define if the activity described here can be translated to human DCs. B cells and plasmacytoid dendritic cells are the primary cells in humans that express TLR9 and respond to CpG stimulation (Hemmi *et al*, 2000; Jarrossay *et al*, 2001; Girsel *et al*, 2002; Hornung *et al*, 2002). The DEC-205 receptor has been recently described to be expressed on human plasmacytoid DCs (Tel *et al*, 2011), and accumulating evidence now suggests that pDCs are efficient in cross-presentation (Hoeffel *et al*, 2007; Di Pucchio *et al*, 2008; Lui *et al*, 2009; Tel *et al*, 2013). In addition, it has recently been reported that human myeloid CD141^+^ DCs produce cytokines in response to CpG-ODN in a whole blood assay (Hémont *et al*, 2013). Thus, in the light of these findings, it would be interesting to analyze the activity of targeted bacteriophages in the human system. Furthermore, immunotherapeutic options based on targeting B cells which also express DEC-205 can be envisaged.

In summary, using mouse BMDCs, we have demonstrated that fd carrier delivered through DEC-205 combines both the functions of immunogenicity and adjuvanticity and thus can bypass the limitations of using anti-DEC-205 monoclonal antibodies and can better exploit the use of this receptor for targeted antigen delivery.

## Materials and Methods

### Purification of bacteriophage particles and Western blot

Wild-type, fdsc-αDEC, fdOVA and fdOVA/sc-αDEC recombinant bacteriophages were purified as described previously (Sartorius *et al*, 2011). The number of copies of pVIII displaying the OVA_257–264_ peptide was estimated by N-terminal sequence analysis of the purified virions and resulted in 15–20% for each phage preparation. The expression of the scFv anti-DEC-205 in the pIII protein of the purified virions was assessed by Western blot analysis as previously described (Sartorius *et al*, 2011). Elimination of LPS was performed by extraction with Triton X-114 (Sigma-Aldrich, Milan, Italy) according to Aida & Pabst, 1990. The final particles were tested for endotoxin using the Limulus Amebocyte Lysate (LAL) Assay (QCL-1000, Lonza, Basel, Switzerland) according to the manufacturer's instructions. The endotoxin levels were < 0.05 EU/ml in all phage preparations.

### Synthesis of antibody–peptide conjugates

The NLDC145 (DEC-205/CD205 ATCC® HB-290™) hybridoma cell line producing the anti-DEC-205 antibody was obtained from the American Type Culture Collection and was maintained in RPMI 1640 medium supplemented with 10% fetal bovine serum (FBS) (Invitrogen Life Technologies, Carlsbad, CA). The NLDC145 antibody was purified from cell culture supernatants using the MAbTrap kit (GE Healthcare & Life Sciences, Milan, Italy) according to the manufacturer's instructions.

Antibodies (5 mg/ml) were reacted in 50 mM potassium phosphate (pH 7.5) with a 30-fold molar excess of N-succinimidyl S-acetylthioacetate (SATA, Thermo Fisher Scientific, Rockford, IL) for 4–6 h at 20°C. Unreacted cross-linking reagents (SATA) were removed by size-exclusion chromatography (Sephadex G25 medium, GE Healthcare, Milan, Italy) against degassed 20 mM potassium phosphate, buffered at pH 7.0 to avoid premature deacetylation on thiols. The resulting antibody was produced with an average of 3–6 blocked thiol groups covalently bound on lysine residues. The degree of linker attachment was estimated by a spectrophotometrical method immediately before subsequent peptide coupling. Briefly, DTNB (Ellman reagent), which has little if any absorbance, reacts with -SH groups on proteins under mild alkaline conditions (pH 7–8) such that the 2-nitro-5-thiobenzoate anion results in an intense absorbance at 412 nm. Since the Ellman reagent is very sensitive to buffer ions, pH and thermochromic effects, an accurate calibration curve using standard cysteine standards was performed using the same buffer as that used for the antibody. Blocking groups were then removed with 20 mM hydroxylamine (Sigma-Aldrich) and a molar excess of OVA_257-264_ peptide containing an N-terminal maleimide (PRIMM, Milan, Italy) was added to approximately 30- to 40-fold molar excess over antibody and allowed to react with the new thiol groups for 2–4 h at 20°C. Excess unreacted peptide and aggregated antibodies were removed by size-exclusion chromatography against PBS. The conjugate addition between thiols and the maleimide moiety is a routine reaction, and a good yield of coupling was confirmed by the absence of any reduced sulfhydryl group on the freshly purified bioconjugate (Ellman), so the product was used as eluted from the column.

### Mice

Six- to eight-week-old female C57BL/6 and ovalbumin (OVA_257–264_)-specific TCR transgenic OT-I mice were purchased from Charles River (Lecco, Italy) and were maintained under specific pathogen-free conditions at the IGB Animal House Facility at CNR, Naples. *MyD88*^*−/−*^, *Tlr4*^*−/−*^ and *Tlr9*^*−/−*^ mice were kindly donated by the University of Messina, Messina, Italy. All mice were on C57BL/6 background. Animals were randomly assigned to treatment groups. All experiments with mice were performed in accordance with European Union Laws and guidelines. All animal studies were approved by our institutional review board, and the animal procedures (i.e., immunization, sacrifice) were performed according to rules approved by the ethics committee (permission n. 137/2006-A).

### Antibodies and fluorescent dyes

Antibodies used for flow cytometry/immunofluorescence, anti-CD8-PE-Cy7 (53-6.7), anti-Vα2-TCR-PE (B20.1) and anti-IFN-γ-PE (XMG1.2), were from Biolegend, San Diego, CA. Anti CD8a-APC (53-6.7) and anti-CD11c-ΑPC (N418) were from eBioscience, San Diego, CA. Anti-CD11c-PE-Cy7 (HL3) was from BD Biosciences. Anti-DEC-205-FITC (NLDC145) was from ACRIS Antibodies GmbH, Herford, Germany.

The rabbit polyclonal antibody anti-LAMP-1 was from Abcam, Cambridge, UK. The rabbit polyclonal antibody anti-EEA-1 was from Cell Signalling, Milan, Italy. The Alexa Fluor 488 and Hoechst were from Life Technologies, Monza, Italy.

### BMDCs

Bone marrow-derived dendritic cells were produced from precursors isolated from the bone marrow of C57BL/6, *MyD88*^*−/−*^, *Tlr4*^*−/−*^ or *Tlr9*^*−/−*^ mice by culturing them with 200 U/ml recombinant murine granulocyte/macrophage colony-stimulating factor (GM-CSF) (Peprotech, NJ, USA) in RPMI 1640 (Lonza) medium supplemented with 10% FBS, 100 U/ml penicillin, 100 mg/ml streptomycin, 1 mM sodium pyruvate and 55 mM 2-mercaptoethanol (all from GIBCO, Milan, Italy). Cells were collected at day 7 of culture and were assayed for dendritic cell phenotype by staining with the monoclonal antibodies anti-CD11c and anti-DEC-205. Staining was performed in phosphate-buffered saline (PBS) containing 0.5% bovine serum albumin (BSA) for 30 min on ice using standard protocols. Data were acquired and analyzed by a BD FACSCanto II flow-cytometer and DIVA software (Becton Dickinson, Fullerton, CA).

### Antigen presentation assay

Bone marrow-derived dendritic cells were incubated overnight in the presence of fdOVA, fdOVA/sc-αDEC phage particles or NLDC:OVA_257-264_ peptide conjugates (all containing from 1 to 0.01 μg/ml OVA_257-264_ peptide). The day after, CD8^+^ OVA_257–264_-specific T cells were purified from the spleen of OT-I mice using the CD8^+^ T-cell isolation kit (Miltenyi Biotec, Calderara di Reno, BO, Italy), according to the manufacturer's instructions. Purified CD8^+^ OT-I T cells were labeled with 5 mM CFSE (Sigma-Aldrich) for 10 min at 37°C. The reaction was quenched with FBS, and cells were washed 2–3 times. DCs were washed extensively to remove free antibody or phage particles and co-cultured with CFSE-labeled OT-I CD8^+^ T cells for 72 h at a DC:T cell ratio of 1:1. After 3 days of co-culture, the CFSE intensity of OT-I T cells was evaluated on CD8^+^-gated cells by flow cytometry using anti-CD8 mAb and a BD FACSCanto II.

### Adoptive transfer and T-cell assays

CFSE-labeled OT-I CD8^+^ T cells (3.5 × 10^6^) were injected intravenously into C57BL/6 recipients. Twenty-four hours later, mice were immunized subcutaneously with fdOVA, fdOVA/sc-αDEC phage particles, NLDC:pOVA or NLDC:pOVA plus 50 μg cytosine-phosphate-guanosine oligodeoxynucleotides (CpG-ODN, InvivoGen, CA, USA) in 1× PBS, all containing the same dose (1.6 μg) of OVA_257-264_ peptide, or with 50 μg of soluble LPS-free ovalbumin protein (ENDOGRADE Ovalbumin, Hyglos GmbH, Germany). As a control, C57BL/6 mice were injected with vehicle alone. After 3 days, splenocytes were isolated and stained with anti-Vα2 and anti-CD8 mAbs. The CFSE fluorescence intensity of adoptively transferred OT-I cells was then evaluated by flow cytometry on CD8^+^Vα2^+^ cells as previously described (Sartorius *et al*, 2011).

IFN-γ-producing CD8^+^ T cells were evaluated by culturing 7 × 10^6^ spleen cells with OVA_257-264_ SIINFEKL peptide (10 mg/ml) for 5 h in the presence of 10 μg/ml brefeldin A (Sigma-Aldrich). Cells were then harvested, and IFN-γ production was evaluated by intracellular staining on gated CD8^+^ T cells using PE-conjugated anti-IFN-γ mAb and Leucoperm fixation and permeabilization kit (AbD Serotec, Oxford, UK).

### Analysis of *in vitro* cytokine production

To analyze IL-6, IL-18 and IFN-α production in the supernatants, DC cultures were derived from bone marrow of C57BL/6 mice and treated for 20 h with 100 μg/ml of the LPS-purified bacteriophage fd particles or anti-DEC-205 antibody NLDC145. As a positive control, supernatant was also collected from DC cultures treated with LPS (0.1 μg/ml, Sigma-Aldrich), Poly I:C (100 μg/ml) or 1 μM CpG-ODN. BMDC cells were also obtained from *MyD88*^*−/−*^, *Tlr4*^*−/−*^ or *Tlr9*^*−/−*^ mice and treated for 20 h with 100 μg/ml of the LPS-purified bacteriophage fd particles. As a positive control, supernatant was also collected from DC cultures treated with LPS or CpG-ODN. IL-6, IL-18 or IFN-α production was measured, according to the manufacturer's instructions, using commercially available ELISA kits: ELISAMAX IL-6 standard set (Biolegend, San Diego, CA), Mouse IL-18 ELISA kit (MBL, Nagoya Aichi, Japan) or VeriKine Mouse Interferon alpha ELISA kit (pbl interferon source, Piscataway, NJ), respectively.

### IL-6 expression analysis by quantitative RT–PCR

C57BL/6, *Tlr9*^*−/−*^ and *MyD88*^*−/−*^ mice were injected intraperitoneally with fdOVA, fdOVAsc-αDEC (100 μg/mouse) or, as a positive control, with LPS (100 μg/mouse) and sacrificed 2 h later. As a negative control, a PBS-injected mouse group was used.

Spleen cells were recovered from each mouse by cutting the spleen into small fragments and incubating with collagenase D type II (Roche Biomedical Laboratories, Burlington, NC) solution (2 mg/ml) for 30 min at 37°C. Digestion was stopped before collection of cell suspensions. The resulting material was then passed through a 70-μm cell strainer, and the recovered cells were subjected to dendritic cell enrichment using magnetic separation with mouse Pan DC microbeads and a MACS separator (Miltenyi Biotec), according to the manufacturer's instructions.

Total RNA was isolated using Tri Reagent (Sigma-Aldrich) according to the manufacturer's instructions. The integrity and quality of RNA were assessed by denaturing agarose gel electrophoresis and by Experion (Bio-Rad, Segrate (MI), Italy). RNA quantification was assessed by spectrophotometry (NanoDrop Technologies). For each sample, 500 ng of total RNA was reverse-transcribed into complementary DNA (cDNA) using the High-Capacity cDNA Reverse Transcription Kit (Life Technologies) according to the manufacturer's protocol. cDNAs were used as template for quantitative real-time polymerase chain reaction assays. The amplification reaction mix contained 1× iTaq™ Universal SYBR® Green Supermix (Bio-Rad), 300 nM of each primer and 50 ng of cDNA (RNA equivalent) as a template. PCR conditions were 95°C for 10 min followed by 40 cycles of 95°C × 30 s, 60°C × 30 s and 72°C × 30 s. Melting curves were generated after amplification. Data were collected using the CFX Connect™ Real-Time PCR Detection System (Bio-Rad); each reaction was performed in duplicate. The relative gene expression was calculated using the 2^−ΔΔCt^ method (Livak & Schmittgen, 2001), using as calibrator PBS-treated mice and β-actin as a housekeeping gene. Primers were designed using Oligo 4.0-s. Sequences of the primers are as follows: *Il6*-for 5′gcctattgaaaatttcctctgg3′; *Il6*-rev 5′ggaaattggggtaggaaggac3′; *Actb*-for 5′ttctttgcagctccttcgtt3′; *Actb*-rev 5′gcacatgccggagccgtt3′.

### OVA_257-264_-specific immune response using *ex vivo* isolated DCs

C57BL/6, *Tlr9*^*−/−*^ and *MyD88*^*−/−*^ mice were injected intraperitoneally with 50 μg of fdOVAsc-αDEC 18 h before sacrifice. Dendritic cells were isolated from the spleen of immunized mice as described above.

CD8^+^ OVA_257-264_-specific T cells were isolated from OT-I mice and labeled with 5 mM CFSE. A total of 100,000 CD8^+^ OT-I purified T cells were added to each well of 96-well flat-bottom plates containing 10,000 DCs isolated from wild-type or KO mice. Proliferation of OVA-specific T cells was determined by flow cytometry after culture at 37°C in 5% CO_2_ for 72 h. Cells were stained with anti-Vα2 mAb before acquisition on FacsCanto II (BD Biosciences). IFN-γ release was assessed by intracellular staining of purified OT-I CD8^+^ T cells co-cultured for 12 h in the presence of 10 μg/ml brefeldin A and 3,300 C57BL/6, *Myd88*^*−/−*^ or *Tlr9*^−/−^ splenic DCs isolated from mice treated as above. The percentage of IFN-γ-producing OT-I CD8^+^ T cells was evaluated on CD8^+^-gated cells by flow cytometry using an anti-IFN-γ mAb and Leucoperm fixation and permeabilization kit.

### RNA-Seq library production, sequencing and data analysis

Bone marrow-derived dendritic cells were plated in the presence of 100 μg/ml of the LPS-purified bacteriophage fd wild-type or fdsc-αDEC for 20 h. Total RNA was extracted as described above. RNA quality was assessed as described in Costa *et al*, 2011. Paired-end libraries (100 × 2 bp) were prepared using the TruSeq RNA Sample Preparation Kit (Illumina), following the manufacturer's instructions. Libraries were sequenced on the Illumina HiSeq2000 NGS platform at high coverage. A total of about 220 million paired-end reads were sequenced. The quality of sequenced reads was assessed using FastQC (http://www.bioinformatics.babraham.ac.uk/projects/fastqc/). TopHat version 2.0.10 (Kim *et al*, 2013) was used to map the reads against the reference mouse genome (mm9) and the RefSeq mouse transcript annotation, with default parameters. RefSeq track was downloaded from Table Browser of UCSC (http://genome.ucsc.edu). Only uniquely mapped reads (about 95% of sequenced reads) were used for further analyses. Coverage files were produced using BEDTools. Visual inspection of reads and coverage files on UCSC Genome Browsers was used to assess the overall quality of the RNA-Seq experiment, and to inspect gene-specific features. Gene expression quantification was performed using Cufflinks (Trapnell *et al*, 2010). The high correlation score of the replicates (mean Pearson's coefficient *r* = 0.95) indicated the high reproducibility of the RNA-Seq experiment. Normalized gene expression values (FPKM) for each condition were used to identify differentially expressed genes using Cuffdiff and CummeRBund (Trapnell *et al*, 2012). Gene ontology and pathway analysis were performed using DAVID (Huang *et al*, 2009). The RNA-Sequencing data from this manuscript have been submitted to the Gene Expression Omnibus (G.E.O.) database (http://www.ncbi.nlm.nih.gov/geo/) and assigned the identifier GSE60231.

### FITC and TRITC conjugation of bacteriophage particles and internalization assay

Fluorescein isothiocyanate conjugation to bacteriophage particles was performed using FITC isomer I (Sigma-Aldrich) as previously described (Sartorius *et al*, 2011). TRITC conjugation of bacteriophages was performed using TRITC from Thermo Fisher Scientific. Briefly, TRITC was added to purified bacteriophages at 6 mg/ml, and the solution was incubated for 2 h at room temperature in the dark. The excess of TRITC was removed by dialysis against 100 mM carbonate/bicarbonate buffer, pH 9.0. The number of fluors per phage was estimated from the UV absorption spectrum of purified and conjugated filamentous phage.

For the internalization assay, BMDCs (10^6^ cells per well) were incubated at 37°C with FITC-conjugated fdsc-αDEC, fdWT bacteriophage particles (100 μg/ml) or NLDC145 antibody. The cells were collected and analyzed by flow cytometry at various time intervals of incubation.

### Vector construction and production of recombinant lentiviral particles

The mouse TLR9-YFP sequence was a kind gift of Prof. Alexander Dalpke, University of Heidelberg, Germany.

The TLR9-YFP sequence was digested with NheI/XhoI and introduced into the same sites of the pTY2CMV-IRES-WPRE plasmid, kindly provided by Dr. A. Cara, ‘Istituto Superiore di Sanità’, Italy, to produce the pTY2-TLR9-YFP plasmid.

Recombinant lentiviral particles were produced as described in Negri *et al*, 2010. Briefly, HEK293T cells (ATCC CRL 1573) were transfected with pTY2-TLR9-YFP, the packaging plasmid pCMVdR8.2 and the vesicular stomatitis virus G glycoprotein (VSV-G) envelope-expressing pMD.G plasmid. The vector-containing supernatant was clarified and concentrated by ultracentrifugation (Beckman Coulter, Fullerton, CA) on a 20% sucrose gradient. Viral pellets were resuspended in 1 × PBS, and viral titer was evaluated by cytofluorimetric analysis of YFP expression after HK293 T-cell infection.

### Transduction with lentiviral vectors and Immunofluorescence

For lentiviral infection, bone marrow mononuclear cells were cultured at 50,000 cells/well in 24-well plates on glass coverslips in the presence of GM-CSF (200 U/ml) and infected with concentrated viral supernatant at MOI 5 at day 1. At day 7, the glass coverslips were collected and incubated with TRITC-conjugated phage particles for 6 h at 37°C. Cells were then washed three times in PBS, fixed in 4% paraformaldehyde (PFA) (Electron Microscopy Sciences, Hatfield, PA) for 10 min, washed three times in PBS and mounted on glass slides using Mowiol (Life Technologies). Nuclei were counterstained with Hoechst. For EEA-1 and LAMP-1 staining, BMDCs at day 7 were collected and seeded onto glass coverslips at 5 × 10^5^ cells/well in 24 well plates. At day 8, the glass coverslips were collected and incubated with TRITC-conjugated phage particles for 6 h at 37°C. Cells were then washed three times in PBS, fixed in 4% PFA for 10 min, permeabilized in 0.05% saponin (Sigma-Aldrich) and counterstained with anti-rat Alexa Fluor-488. Following labeling, coverslips were washed three times in PBS and mounted on glass slides.

### Confocal fluorescence microscopy, image processing and co-localization analysis

Immunofluorescence microscopy and quantitative image analysis were performed as described (Daniele *et al*, 2008; Vicinanza *et al*, 2011). Images were acquired on a Zeiss LSM 700 confocal laser scanning microscope, using a 65× oil-immersion objective and Zeiss’ ZEN imaging software.

The experiments were repeated twice, and representative images are shown. The level of co-localization (i.e., LAMP1-fdWT, LAMP1-fdsc-αDEC, TLR9-fdWT, TLR9-fdsc-αDEC) was calculated by acquiring confocal serial sections from about 30 cells, exported in TIFF format and processed as previously described (Daniele *et al*, 2008; Vicinanza *et al*, 2011).

**The paper explained**

**Problem**

A promising immunotherapeutic approach relies on the direct targeting of dendritic cells (DCs) by coupling antigens to antibodies able to recognize the DEC-205 receptor which is specifically expressed by DCs. However, it has been widely reported that this delivery is efficient only if associated with the administration of maturation stimuli for DCs, such as anti-CD40 or Poly I:C, and it is known that administration of these adjuvants may have side effects. In this context, we previously reported the induction of protective CD8^+^ T-cell responses by immunizing with filamentous bacteriophage fd particles displaying an anti-DEC-205 single-chain variable fragment (fdsc-αDEC) in the absence of adjuvants or maturation stimuli for dendritic cells.

**Results**

In this study, we provide evidence for the fdsc-αDEC mechanism of action by showing that antigen delivery by filamentous fd bacteriophage to dendritic cells via DEC-205 in the absence of adjuvants determines the up-regulation of several gene pathways involved in the immune response such as TLR genes, co-stimulatory genes and genes coding for pro-inflammatory cytokines. In addition, we observed that the production of these cytokines was MYD88 mediated and TLR9 dependent. Finally, we demonstrated that filamentous bacteriophages containing a single-strand DNA genome rich in CpG motifs are addressed to the late endosome compartments where they co-localize with TLR9 molecules, present in these compartments in their active form. We conclude that this interaction enhances the immunogenicity of the antigenic determinants displayed on the phage scaffold.

**Impact**

These findings make filamentous bacteriophage fd particles a valuable tool for immunization without administering exogenous adjuvants. Bacteriophage production is economically advantageous, and all available evidence indicates that administration of different strains of bacteriophages is harmless to humans. Thus, this study may be relevant concerning the possibility to use this system in humans.

### Statistical analysis

Results are expressed as the mean ± SD. All data were tested with the Kolmogorov–Smirnov test for normal distribution. The statistical significance of differences between experimental groups was calculated using the unpaired two-tailed Student's *t*-test with equal variance. All exact *P*-values are indicated in each figure. Differences were considered statistically significant when *P*-values were < 0.05.

## Supporting Information

Supplementary Figures

Review Process File
