# Supplementary material for: Antigen delivery by filamentous bacteriophage fd displaying an anti-DEC-205 single-chain variable fragment confers adjuvanticity by triggering a TLR9-mediated immune response
Source: EMBO Mol Med. 2015 Apr 17;7(7):973–88. doi: 10.15252/emmm.201404525 (PMC4520660; doi:10.15252/emmm.201404525)
Supplement: Supplementary file 1 [file emmm0007-0973-sd1.pdf]

Table of contents:

Supplementary Figure S1
Supplementary Figure S2
Supplementary Figure S3

Supplementary Figure S1

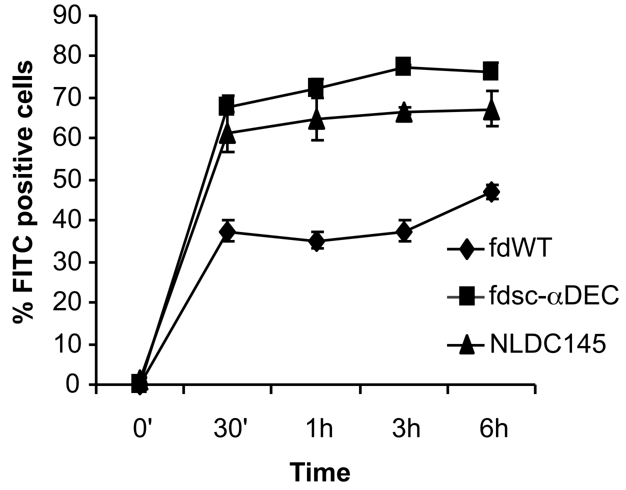

Supplementary Figure S1. Time-course analysis of fdsc-αDEC internalization by DCs. BMDCs were incubated with FITC-conjugated fd wild type (fdWT) or fdsc-αDEC bacteriophages or with FITC-anti-DEC-205 antibody (NLDC145) at 37°C for the indicated time. Cells were then stained for CD11c and analysed by flow cytometry. Percentage of FITC-positive CD11c+ gated dendritic cells is reported. Average values of two experiments are displayed.

Supplementary Figure S2

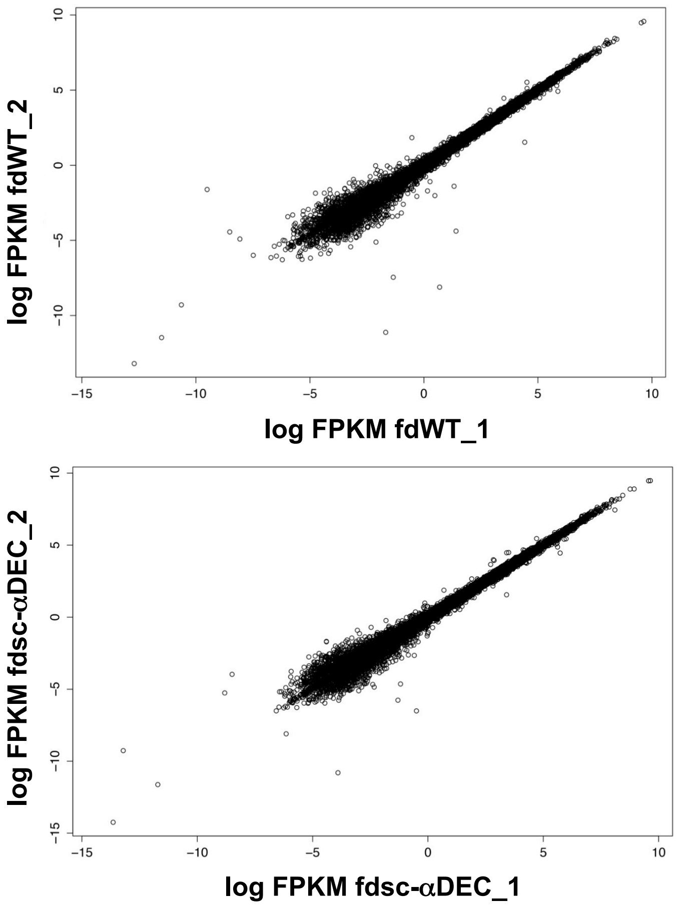

Supplementary Figure S2. FPKM correlation between the technical replicates. A) log FPKM (Fragments Per Kilobase of transcript and Million of mapped reads), for fd wild type (fdWT) replicates 1 and 2; **B)** log FPKM for fdsc-αDEC replicates 1 and 2.

Supplementary Figure S3

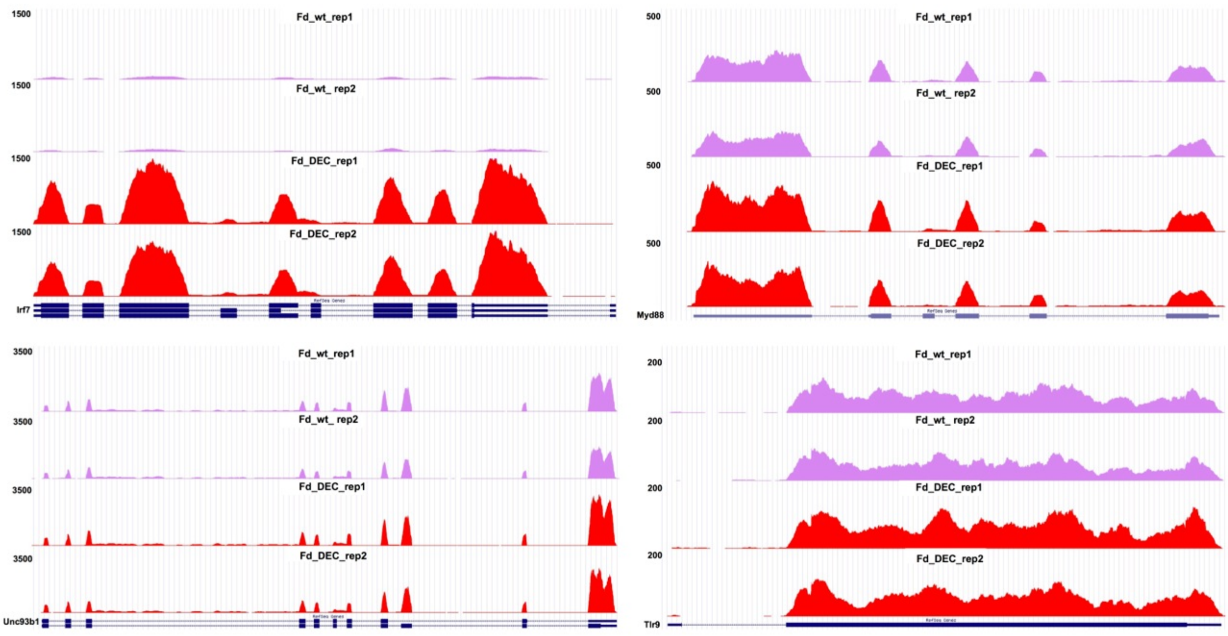

Supplementary Figure S3. RNA-Seq data panel for *Irf7, MyD88, Unc93b1 and Tlr9* genes. Colored peaks indicate the reads' coverage across the RefSeq genes (indicated by the blue boxes). In particular, violet and red peaks refer to RNA-Seq coverage for fd wild type (Fd_wt) and fdsc-αDEC (Fd_DEC) replicates, respectively. Relative coverage values are indicated on the left of each panel.
