# Supplementary material for: Antigen delivery by filamentous bacteriophage fd displaying an anti-DEC-205 single-chain variable fragment confers adjuvanticity by triggering a TLR9-mediated immune response
Source: EMBO Mol Med. 2015 Apr 17;7(7):973–88. doi: 10.15252/emmm.201404525 (PMC4520660; doi:10.15252/emmm.201404525)
Supplement: Supplementary file 2 [file emmm0007-0973-sd2.pdf]

Antigen delivery by filamentous bacteriophage fd displaying an anti-DEC-205 single-chain variable fragment confers adjuvanticity by triggering a TLR9-mediated immune response

Rossella Sartorius, Luciana D'Apice, Maria Trovato, Fausta Cuccaro, Valerio Costa, Maria Giovanna De Leo, Vincenzo Manuel Marzullo, Carmelo Biondo, Sabato D'Auria, Antonella De Matteis, Alfredo Ciccodicola and Pierguiseppe De Berardinis

Corresponding author: Piergiuseppe De Berardinis, I.B.P., C.N.R.

Review timeline:

Submission date:	08 August 2014
Editorial Decision:	19 September 2014
Appeal:	23 September 2014
Additional Correspondence:	29 September 2014
Revision received:	22 January 2015
Editorial Decision:	10 March 2015
Revision received:	19 March 2015
Accepted:	24 March 2015

Transaction Report:

(Note: With the exception of the correction of typographical or spelling errors that could be a source of ambiguity, letters and reports are not edited. The original formatting of letters and referee reports may not be reflected in this compilation.)

1st Editorial Decision 19 September 2014

Thank you for the submission of your manuscript to EMBO Molecular Medicine. We have now heard back from the three Reviewers whom we asked to evaluate your manuscript.

We are very sorry that it has taken longer than usual to get back to you on your manuscript. In this case, also due to the holiday season, we experienced significant difficulties in securing three expert and willing Reviewers. Further to this the evaluations were delivered with some delay.

As you will see, while Reviewer 2 appears more supportive of your study, Reviewers 1 and 3 point to significant and fundamental issues that, I am afraid, preclude publication of the manuscript in EMBO Molecular Medicine. I will not discuss each point in detail as they are clearly stated.

In brief, the main concerns raised by Reviewers 1 and 3 are centred on the lack of a convincing in vivo proof of concept, which would be required to lend translational interest to the findings. Specifically, the two Reviewers note that further in vivo experimentation in MyD88 and TLR9 KO mice would be needed. Furthermore, it is essential to test whether scFv anti-human DEC-205 phages activate the different DC populations, which in turn would require the generation of a new bacteriophage.

Reviewer 2, while more supportive and in addition to other issues, challenges the safety premise presented in the manuscript and notes missing controls in the cytokine release experiments with phage-stimulated BMDCs. S/he also mentions that at this stage the manuscript would appear more suited to a specialist rather than a broad interest venue.

Given these fundamental concerns and the overall lack of enthusiasm by the Reviewers, I have no choice but to return the manuscript to you at this stage. In our assessment it is not realistic to expect to be able to address these issues experimentally and to the satisfaction of the Reviewers in a reasonable time frame. Especially considered that experimentation would have to include route of administration, toxicity and long-term evaluations.

I wish to add that, considered the potential interest of these findings, we would have no objection to consider a new manuscript on the same topic if at some time in the near future you have obtained data that would considerably strengthen the message of the study and address the Reviewers' concerns. Please consider, however, that if you were to send a new manuscript this would be treated as a new submission rather than a revision and would be reviewed anew.

I am sorry to have to disappoint you at this stage. I hope that the Reviewers' comments will be helpful in your continued work in this area.

***** Reviewer's comments *****

Referee #1 (Comments on Novelty/Model System):

As indicated in the comments to the authors, in vivo experiments in MyD88 ko and TLR9 ko mice need to be performed in order to verify the relevance of the identified mechanism in the immunogenicity.

Referee #1 (Remarks):

The manuscript by Sartorius et al. investigates the molecular mechanism responsible for DC maturation triggered by a recombinant filamentous bacteriophage displaying a scFv anti-DEC-205, described in a previous paper (Sartorius et al. EJI 2011). In the current manuscript the authors show that the DEC-205 targeting phages are delivered to the late endosome/lysosome compartment of BMDC, where TLR9 is engaged by the phage CpG rich DNA leading to DC maturation. Although this work identifies a possible mechanism responsible for the efficacy of DEC-205 targeting phage particles as immunizing system, in its current version the work does not show sufficient data when compared to previous publications and is not suitable for publication in EMBO Molecular Medicine. In particular no in-vivo experiments are presented to support the relevance of the described pathway for the immunogenicity of the scFv anti-DEC-205 displaying phages. Moreover, no experiments with human cells were performed to verify the importance of the identified mechanism in the activation of human DC populations for which expression of TLR9 is different compared to the mouse system. This is crucial for the application of the DEC-205 targeting phage as a delivery system for vaccination in humans, as the authors propose.
Points to be addressed:
1) The author should perform immunization experiments in MyD88ko and TLR9ko mice with the DEC-205 targeting as well as the WT phages, to demonstrate that the increased immunogenicity of anti-DEC-205 phages is dependent on the TLR9-MyD88 pathway;
2) The author should test the ability of scFv anti-human DEC-205 phages to activate the different human DC populations (in particular the CD141+ population and the pDC). This may of course require production of a new recombinant bacteriophage expressing a scFV recognizing the human DEC-205, but it is critical to generate data in support of the proposed application of this technology for human use;
3) Figure 1B, C and D: it is unexpected that the anti-DEC-205 conjugated OVA peptide does not activate OT-I cells as shown in a previous publication (Bonifaz et al., JEM 2004). How do the authors explain this result? Addition of an adjuvant, such as CpG, could help to test the immunogenicity of the conjugated peptide. Some controls are also missing in these experiments: immunization with the unconjugated peptide and with LPS free ovalbumin.
4) Figure 1 F and G: cytokine production induced by Poly I:C and CpG should be shown here;
5) Figure 3: BMDC from WT mice need to be tested in parallel to KO cells in the same experiment;
6) Figure 5C: image on the right (merge) seems identical to image in the middle (red fluorescence) not a merged image;
7) Page 4 lane 10: in the paper cited (Bonifaz et al., JEM 2004) immunogenicity of the anti-DEC205 conjugated OVA is increased by addition of anti-CD40, not by CpG or Poly I:C as indicated in the

text.
8) Page 4 lanes 22-23: "Phage administration has proven to be safe (Reardon, 2014)": this statement is misleading since no human test with this type of recombinant bacteriophages as a vaccine delivery system has been performed so far. The cited reference regards the use of phages to treat bacterial infections and is not appropriate here.

Referee #2 (Comments on Novelty/Model System):

The targeted bacteriophage antigen delivery system is innovative. In mice, the targetting appears effective at increasing immune responses. Until it is tested in humans, we do not know whether it will prove similarly immunogenic, and we do not know if it will be well tolerated and safe. Nevertheless, mouse experiments are a good early step.

Referee #2 (Remarks):

This manuscript describes comparisons between stimulation of mouse bone marrow-derived dendritic cells (BMDCs) by bacteriophage fd (in some cases bearing an ovalbumin-derived antigen) with and without targeting by a single chain variable fragment that binds the DC-specific antigen DEC-205. The experiments indicate that OVA antigen-bearing and DEC-205-targeted bacteriophage more efficiently stimulate anti-OVA specific T cells than their non-targeted equivalents when loaded into BMDCs. The targeted OVA-displaying bacteriophage are more efficient at eliciting CD8+, OVA-specific T cell proliferation and interferon gamma production when used as immunogens than non-targeted bacteriophage. Targeted fd phage are also more efficient at inducing IL-6, IL-18, and IFN-gamma from exposed DCs than their non-targeted equivalents.

The authors probe the cause of these differences by comparing the RNA expression profiles of exposed DCs and find that a number of pathways involved in innate immunity and cytokine production are differentially up-regulated by the targeted bacteriophage. Based on the pattern of up-regulation and the presence of a single-stranded, non-methylated DNA genome in the phage, the authors hypothesize that the TLR9 and MyD88 pathways may be involved in the stimulated cytokine release, and they confirm this hypothesis for IL6 and IFN-alpha release by stimulating DCs from MyD88 and TLR9 knock-out mice. Following up, they find that targeting of the bacteriophage with anti-DEC-205 increases their localization to DC late endosomal compartments and their co-localization in with TLR9, which appears to be upregulated in the DCs stimulated by the targeted phage.

The potential significance of the study relates to the use of ex-vivo DC stimulation for immunotherapy. Antigen targeting to DCs with anti-DEC 205 only stimulates the DCs if adjuvants are co-administered. Using targeted bacteriophage to deliver the antigens combines the adjuvant with the antigen. The authors suggest that there are safety advantages to this approach.

The findings of increased DC cell stimulation and immunogenicity with targeted bacteriophage are convincing. The transcriptome analysis correlates nicely with the phenotypic findings and is backed up by the findings in DCs from knock-out mice. The co-localization of the targeted phage with TLR9 implies an interaction (whether direct or indirect) may take place in late endosomes, and an adjuvant effect of the phage genome is indeed possible, though not proven. For these reasons, the manuscript could be of interest to readers with an interest in antigen delivery, immunotherapy, and mechanisms of adjuvant activity.

As detailed below, there are significant weaknesses in the
writing of the manuscript. The safety concerns from the use of added adjuvants take the form of vague implications rather than data-based findings, and assertions of the known safety of bacteriophage therapy are exaggerated. As these proposed safety differences, which are not addressed experimentally in the manuscript, are provided as a chief justification for the study, this issue weakens the manuscript. The descriptions of key experiments in the Results section contain too little information to follow (although the Materials and Methods are better written). There are problems in figure labeling, and the manuscript needs extensive editing for English. The experiments with DCs from knock-out mice appear to be missing a well-matched control from non-knock-out mice.

The length of the manuscript is appropriate for the amount of data.

Major specific comments.

1. The title and other parts of the manuscript state that the display of the antigen of interest in bacteriophage bypasses the need for adjuvants. One common definition of an adjuvant is "a substance that enhances the body's immune response to an antigen." In this sense, the targeted phage particle does not bypass the need for an adjuvant. Rather, the targeted phage particle is itself an adjuvant, combined with an antigen. Do the authors see an advantage of combining an antigen and adjuvant over adding an adjuvant to an antigen?

2. The manuscript should be edited by a fluent English speaker.

3. In the Results section corresponding to Fig. 1, Suppl. Fig. S1, and Suppl. Fig. S2, the experiments are not described clearly enough to understand what has been done. It was necessary to flip back and forth between Results, Materials and Methods, and Figure legends repeatedly in an attempt to deduce the basic outline of the experiments that generated the results. Although there should not be extensive repetition of the Material and Methods and Figure Legend in the Results section, the Results section should contain enough information to follow the experimental logic. For example, it should be clearly stated whether the particles in each experiment contain the OVA peptide, whether the antigens are being used to immunize animals or stimulate cells in culture, and the origin of cells that are analyzed in vitro.

4. Introduction. A statement is made that "Phage administration has proven to be safe..." Impact section. A statement is made that "all available evidences indicate that their administration is entirely harmless to humans." These are overly broad, blanket statements that go beyond levels of certainty that can be provided even for medications that have been studied in very large subject populations. All medical interventions have some risk, and our knowledge of the level of risk depends on the level of safety testing or monitoring that has been done. A more defensible statement should be substituted, one that gives some sense of how many subjects have been observed for adverse events, with what control groups, and with what resulting adverse event profile.

5. Description of the Suppl. Fig. S1 experiment given in the Results section notes rapid internalization of the targeted bacteriophage. However, the data show no difference in the speed of internalization of the various preparations. It is the peak level of internalization that is seen to vary with formulation, not the speed of internalization.

6. Fig. 3 shows the release of IL-6 or IFN-alpha from BMDCs of various knock-out mice upon stimulation with targeted or non-targeted bacteriophage. The Figure contains no control testing BMDCs from mice without a gene knock-out. The text in the Results section refers to Fig. 1e as a comparator. Was the experiment depicted in Fig. 1e conducted as a control for the experiments depicted in Fig. 3, with the only difference being the use of mice without the gene knock-outs in the control? If so, the data should be shown in Fig. 3, rather than in Fig 1, which appears to include panels depicting a disparate set of experiments. If not, the experiments in Fig. 3 lack a key control.

7. Based on the inhibition of the TLR9 signaling pathway and the co-localization of the TLR9 and the targeted particles in late endosomes of BMDCs, the authors hypothesize (p. 11 and Abstract) that the targeted particle interacts with TLR9. It should be pointed out that the paper does not provide biochemical evidence indicating a direct interaction between the targeted particles and TLR9. The inferred interaction could be indirect.

8. The Paper Explained section, p. 20. The authors write that alternatives to co-stimulation of DCs with anti-CD40 or poly I:C are needed for DC-targeted immunotherapy because "it is known that administration of these adjuvants may have far reaching consequences." This is a vague but alarming statement, implying but not explicitly stating a safety concern. What are the far reaching consequences that the use of these adjuvants for immunotherapy "is known" to have, and what is the evidence upon which this conclusion is based?

Minor specific comments

1. "Vehiculated" is not standard terminology. Please define. Would "targetted by" or "delivered by" be reasonable substitutes?

2. Introduction. States that DEC-205 lacks the "consensus amino acid sequences required for calcium or carbohydrate binding." This immediately raises the question, does DEC-25 bind calcium or carbohydrates? The description that it is a C-type lectin receptor implies that it does bind carbohydrates, but this is ambiguous. If it does bind calcium and carbohydrates, then the consensus amino acid sequences must not be required for binding. This confusing description should be clarified.

3. Suppl. Fig. S2. The x-axis needs a label. Perhaps the x and y axes have the same units, each corresponding to a separate replicate experiment so that correlation is shown.

4. Fig. 2. Define "MA" in "MA plot" for Fig. 2a. In multiple panels, symbols and text are too small to read.

5. In Fig. 2c, the color key x-axis is labeled as "value." This is vague - what value is being depicted?

6. One of the pathways that are shown as up-regulated in Fig. 2b is the "systemic lupus erythematosus" pathway. What is meant by this? Some discussion is merited to address the immediate questions that come to mind regarding any potential safety implications of the finding.

7. To what level was LPS eliminated during the preparation of the bacteriophage particles?

Referee #3 (Remarks):

In this paper, the authors investigated the molecular and cellular mechanisms that occurs when filamentous bacteriophages (fd) are used to deliver antigenic determinants to dendritic cells in vitro and in vivo. To this aim, they have used fd particles displaying the anti-DEC-205 scFv fragment to target these particles to DEC205-expressing DCs. In a first set of experiments, the authors compared the ability of fdOVA-DEC and fdOVA particles to induce the proliferation of OVA-specific OTI TCR transgenic T cells in vitro and in vivo. Results convincingly showed that fdOVA/sc-DEC were more efficient that fdOVA to induce OTI proliferation and their differentiation into cytokine-secreting cells. Further experiments, including whole transcriptomic analysis of fd-treated BMDC, showed that targeting fd particles to DEC205 resulted in DC activation, and more specifically in the production of inflammatory cytokines. In further experiments, the authors used both genetic and imaging tools to convincingly show that TLR-9 played a critical role in the sensing of DEC205-targeted fd by DCs.

Major comment:
While BMDCs have been widely used to investigate the mechanisms by which DCs are activated, the DCs that are targeted by fdOVA-DEC in vivo could behave quite differently from BMDC treated with fdOVA-DEC in vitro. To address this issue, the authors could possibly inject C57BL/6 or TLR-9-KO mice with fdOVA-DEC, sort DCs by FACS or using magnetic beads, and monitor the expression of inflammatory cytokines by quantitative PCR.

Appeal 23 September 2014

Thank you for sending the decision letter. I understand your effort in obtaining reviews and of course accept the apologies about the delay. I found many of the criticisms raised by reviewer 2 and 3 helpful to improve the manuscript and I am willing to follow them and perform in vivo experiments in the mouse system, in order to make a better paper.

In contrast, let me say that I found excessive the request by ref 1 (I do not see a similar request in the criticisms raised by ref 3) to consider the experiments on the human system as a prerequisite for publication on your journal. EMBO Mol Med publishes many papers based exclusively on mice models and I have enquired before application on this issue. I agree that the work on human cells

would be informative and interesting. However, it would be another work that needs the engineering of new constructs, a considerable amount of time to be performed and overall, needs to be financed through a specific application once the proofs of concept are provided such as published papers on the mouse system.

I consider that as also suggested by referees 2 and 3 the validation of our system in vivo in mouse should provide strong evidences for publication of our results, and I believe as stated by ref 2 that "the manuscript could be of interest to readers with an interest in antigen delivery, immunotherapy, and mechanisms of adjuvant activity". Indeed, I thought after the pre-submission enquiry that this was also an interest for the readers of EMBO Mol Med.

For this reason, and taking into account your consideration on the potential interest of our findings and that you would have no objection to consider a new manuscript on the same topic, I am asking your advice on the possibility to submit again (as a new submission) this manuscript to your journal once we will be able to perform in vivo in mice the experiments suggested by rev 2 and 3.

Additional Correspondence 29 September 2014

Thank you for your letter regarding our recent decision on your manuscript entitled " Antigen delivery by filamentous bacteriophage fd displaying anti-DEC-205 single-chain variable fragment bypasses the need of adjuvants by triggering TLR9 in late endosomes" and your continued interest in publishing your work EMBO Molecular Medicine.

I do understand your request to reconsider our initial decision and I thank you for highlighting the key aspects of the manuscript. However, please note that in our initial assessment, we had already considered the points you raise. Nevertheless, we have now re-discussed your manuscript without prejudice, including with an external advisor.

Please allow me to first clarify a few issues arising from your rebuttal. Clinical relevance and/or translational potential are of fundamental importance for EMBO Molecular Medicine. Specific circumstances, settings, prior knowledge and other factors may dictate however, and based on the Reviewers' expert opinion, that experimentation on human based systems may be required; in other cases a Drosophila-based model may be sufficient because there is no acceptable mammalian equivalent. Obviously each manuscript has its own merits and cannot be evaluated in a comparative fashion. The second issue is that an expression of interest after a pre-submission enquiry cannot of course be binding. In this case, and as you state, I actually confirmed our interest in my decision letter.

That said, and after discussion with the external advisor, if you address all the other concerns expressed by the Reviewers, including obtaining mechanistic proof of principle by in vivo experimentation with Myd88 and TLR9 KO mice, we would be happy to reconsider a revised manuscript, without the specific requirement to perform further experimentation on human cells.

Please let me know if you wish to move forward with your manuscript as suggested above.

I look forward to seeing a revised form of your manuscript.

1st Revision - authors' response 22 January 2015

Authors' Point-by-Point Reply to Reviewer Comments:

Referee #1

1) The author should perform immunization experiments in MyD88ko and TLR9ko mice with the DEC-205 targeting as well as the WT phages, to demonstrate that the increased immunogenicity of anti-DEC-205 phages is dependent on the TLR9-MyD88 pathway;

These experiments have been performed and are reported in figure 3. They are described in results (page 8 of the revised text).

2) The author should test the ability of scFv anti-human DEC-205 phages to activate the different human DC populations (in particular the CD141+ population and the pDC). This may of course require production of a new recombinant bacteriophage expressing a scFV recognizing the human DEC-205, but it is critical to generate data in support of the proposed application of this technology for human use;

This issue is of undoubted interest and as stated by the referee, would require the production of a new recombinant bacteriophage. We hope to address it in a future work.

3) Figure 1B, C and D: it is unexpected that the anti-DEC-205 conjugated OVA peptide does not activate OT-I cells as shown in a previous publication (Bonifaz et al., JEM 2004). How do the authors explain this result?
Indeed a significant proliferative activity using NLDC:pOVA (anti-DEC205 mAb conjugated to OVA peptide) was observed in the adoptive transfer experiment (Figure 1B, p=0.039 vs control), while no IFN-g production was observed (Figure 1 C). These results are in agreement with the data previously reported by Bonifaz et al (JEM, 2004).
With data reported in Figure 1, we would like to emphasize that, in an experimental setting characterized by the absence of exogenously added adjuvants, the proliferative activity induced by NLDC:pOVA is inferior to the proliferative activity induced by fd bacteriophage DEC-205-targeted particles displaying the OVA peptide.

Addition of an adjuvant, such as CpG, could help to test the immunogenicity of the conjugated peptide.
It has been previously described that addition of exogenous adjuvants as CpG increases the immunogenicity of antigens delivered by anti-DEC-205 mAb. In this manuscript we focused our attention on the use of the fd carrier as being able to confer a strong immunogenicity to the displayed antigenic determinants without the addition of exogenous adjuvant.

Some controls are also missing in these experiments: immunization with the unconjugated peptide and with LPS free ovalbumin.
We now report in Figure 1 the response to LPS free Ovalbumin (50 mg/ml), as a control of OT-I proliferative capability and specificity. In this context ,we reckoned that the use of unconjugated synthetic peptide as control was reduntant and we did not use it.

4) Figure 1 F and G: cytokine production induced by Poly I:C and CpG should be shown here
In this figure we now report in all panels the use of LPS as a positive control. We consider redundant to report the use of Poly I:C as positive control. Concerning CpG, we report it as control in figure 3 (where response to MyD88$^{-/-}$ and TLR9$^{-/-}$ BMDCs in comparison to wild type BMDCs is illustrated).

5) Figure 3: BMDC from WT mice need to be tested in parallel to KO cells in the same experiment;

We have modified figure 3 according to Referee's suggestion.

6) Figure 5C: image on the right (merge) seems identical to image in the middle (red fluorescence) not a merged image;

This figure has been already amended in the previous submission.

7) Page 4 lane 10: in the paper cited (Bonifaz et al., JEM 2004) immunogenicity of the anti-DEC205 conjugated OVA is increased by addition of anti-CD40, not by CpG or Poly I:C as indicated in the text.

We agree with the Referee that we did only provided the reference concerning the use of anti-CD40. We now provide the missing references concerning the use of Poly I:C or CpG to induce the immunogenicity of antigen conjugated to anti-DEC-205 mAb.

8) Page 4 lanes 22-23: "Phage administration has proven to be safe (Reardon, 2014)": this statement is misleading since no human test with this type of recombinant bacteriophages as a vaccine delivery system has been performed so far. The cited reference regards the use of phages to treat bacterial infections and is not appropriate here.

The statement has been modified as follows:
Bacteriophages only infect and multiply with their specific host and currently the therapeutic use of bacteriophages is back on the agenda as bacterial resistance to antibiotics becomes widespread (Reardon, 2014). It should be mentioned that the use of the bacteriophage ΦX174 to assess specific antibody responses in patients with immunodeficiencies has been reported for many years, and is considered a safe, well-tolerated and clinically useful method (Smith et al, 2014). In theory, the administration of filamentous bacteriophage fd in humans should be also considered safe, even if no human tests with this type of recombinant bacteriophage as a delivery system have been performed so far.

Referee #2

Major specific comments.

1. The title and other parts of the manuscript state that the display of the antigen of interest in bacteriophage bypasses the need for adjuvants. One common definition of an adjuvant is "a substance that enhances the body's immune response to an antigen." In this sense, the targeted phage particle does not bypass the need for an adjuvant. Rather, the targeted phage particle is itself an adjuvant, combined with an antigen.

According to referee criticism we changed the title as follows:
"Antigen delivery by filamentous bacteriophage fd displaying an anti-DEC-205 single-chain variable fragment confers adjuvanticity by triggering a TLR9-mediated immune response".

Do the authors see an advantage of combining an antigen and adjuvant over adding an adjuvant to an antigen?

We have not performed this experiment. It is generally thought that avoiding the requirement for adjuvants would be an advantage in clinical application and we reckon that use of carriers with adjuvant property and thus not requiring administration of other exogenous adjuvants may also represent an advantage.

2. The manuscript should be edited by a fluent English speaker.

The revised manuscript has been now edited by a native English speaker.

3. In the Results section corresponding to Fig. 1, Suppl. Fig. S1, and Suppl. Fig. S2, the experiments are not described clearly enough to understand what has been done. It was necessary to flip back and forth between Results, Materials and Methods, and Figure legends repeatedly in an attempt to deduce the basic outline of the experiments that generated the results. Although there should not be extensive repetition of the Material and Methods and Figure Legend in the Results section, the Results section should contain enough information to follow the experimental logic. For example, it should be clearly stated whether the particles in each experiment contain the OVA peptide, whether the antigens are being used to immunize animals or stimulate cells in culture, and the origin of cells that are analyzed in vitro.

According to referee suggestion we rewrote these sections hoping to have made them more clear.

4. Introduction. A statement is made that "Phage administration has proven to be safe..." Impact section. A statement is made that "all available evidences indicate that their administration is entirely harmless to humans." These are overly broad, blanket statements that go beyond levels of certainty that can be provided even for medications that have been studied in very large subject populations. All medical interventions have some risk, and our knowledge of the level of risk depends on the level of safety testing or monitoring that has been done. A more defensible statement

should be substituted, one that gives some sense of how many subjects have been observed for adverse events, with what control groups, and with what resulting adverse event profile.

These statements have been modified either in the Introduction or in the Impact section.

5. Description of the Suppl. Fig. S1 experiment given in the Results section notes rapid internalization of the targeted bacteriophage. However, the data show no difference in the speed of internalization of the various preparations. It is the peak level of internalization that is seen to vary with formulation, not the speed of internalization.

We agree with this criticism and amended the description of suppl figure S1 (pages 5-6 of the text).

6. Fig. 3 shows the release of IL-6 or IFN-alpha from BMDCs of various knock-out mice upon stimulation with targeted or non-targeted bacteriophage. The Figure contains no control testing BMDCs from mice without a gene knock-out. The text in the Results section refers to Fig. 1e as a comparator. Was the experiment depicted in Fig. 1e conducted as a control for the experiments depicted in Fig. 3, with the only difference being the use of mice without the gene knock-outs in the control? If so, the data should be shown in Fig. 3, rather than in Fig 1, which appears to include panels depicting a disparate set of experiments. If not, the experiments in Fig. 3 lack a key control.

According to these suggestion we amended figure 3 and inserted the control testing BMDCs from mice without a gene knock-out.

7. Based on the inhibition of the TLR9 signaling pathway and the co-localization of the TLR9 and the targeted particles in late endosomes of BMDCs, the authors hypothesize (p. 11 and Abstract) that the targeted particle interacts with TLR9. It should be pointed out that the paper does not provide biochemical evidence indicating a direct interaction between the targeted particles and TLR9. The inferred interaction could be indirect.

We agree with this criticism and now we point out it in the text (page 9) as follows:
" These data of co-localzation indirectly suggest that phage particles, containing a single strand DNA genome rich in CpG motifs, when delivered via DEC-205, are able to intercept and trigger the active TLR9 innate immune receptor into the late endolysosomal compartments, and thus to enhance the immunogenicity of the displayed antigenic determinants."

8. The Paper Explained section, p. 20. The authors write that alternatives to co-stimulation of DCs with anti-CD40 or poly I:C are needed for DC-targeted immunotherapy because "it is known that administration of these adjuvants may have far reaching consequences." This is a vague but alarming statement, implying but not explicitly stating a safety concern. What are the far reaching consequences that the use of these adjuvants for immunotherapy "is known" to have, and what is the evidence upon which this conclusion is based?

We changed the sentence and provided the references concerning the side effects caused by administration in humans of Poly I:C or anti-CD40. In particular, intravenous injections of Poly I:C have been associate with intolerable effects at the doses found to be effective in mice (Cornell et al 1976; Levine et al 1978). Similarly, repeated intravenous injections of agonistic CD40 antibody have been reported to cause splenomegaly (Cairing et al 2005).

Minor specific comments

1. "Vehiculated" is not standard terminology. Please define. Would "targetted by" or "delivered by" be reasonable substitutes?

We changed the terminology.

2. Introduction. States that DEC-205 lacks the "consensus amino acid sequences required for calcium or carbohydrate binding." This immediately raises the question, does DEC-25 bind calcium or carbohydrates? The description that it is a C-type lectin receptor implies that it does bind carbohydrates, but this is ambiguous. If it does bind calcium and carbohydrates, then the consensus amino acid sequences must not be required for binding. This confusing description should be

clarified.

It is not yet known which carbohydrate is recognized by DEC-205. Thus the question is open. We tried to clarify better this concept in the introduction.

3. Suppl. Fig. S2. The x-axis needs a label. Perhaps the x and y axes have the same units, each corresponding to a separate replicate experiment so that correlation is shown.

We added the label, and as you have remarked x and y have the same units each corresponding to a separate replicate experiment.

4. Fig. 2. Define "MA" in "MA plot" for Fig. 2a. In multiple panels, symbols and text are too small to read.

"MA" has been defined and larger characters are now used.

5. In Fig. 2c, the color key x-axis is labeled as "value." This is vague - what value is being depicted?

The value "FPKM" has been specified and labeled.

6. One of the pathways that are shown as up-regulated in Fig. 2b is the "systemic lupus erythematosus" pathway. What is meant by this? Some discussion is merited to address the immediate questions that come to mind regarding any potential safety implications of the finding.

We have reported the most enriched biological pathways identified from KEGG database. The lupus pathway contains genes related to inflammation such as genes of costimulus and cytokines which are also present in other pathways.

7. To what level was LPS eliminated during the preparation of the bacteriophage particles?

This has been now reported in methods (page 14)

Referee #3 (Remarks):

Major comment:
While BMDCs have been widely used to investigate the mechanisms by which DCs are activated, the DCs that are targeted by fdOVA-DEC in vivo could behave quite differently from BMDC treated with fdOVA-DEC in vitro. To address this issue, the authors could possibly inject C57BL/6 or TLR-9-KO mice with fdOVA-DEC, sort DCs by FACS or using magnetic beads, and monitor the expression of inflammatory cytokines by quantitative PCR.

We are grateful to Referee # 3 for suggesting this important experiment that we have performed. The results are reported at page 8 and in figure 3. We demonstrate that DCs sorted from the spleen of $MyD88^{-/-}$ *and* $TLR9^{-/-}$ *mice injected with fdOVA-DEC behave as* $MyD88^{-/-}$ *and* $TLR9^{-/-}$ $BMDC_S$ *treated in vitro with fdOVA-DEC.*

2nd Editorial Decision 10 March 2015

Thank you for the submission of your revised manuscript to EMBO Molecular Medicine. We have now received the enclosed reports from the referees that were asked to re-assess it. As you will see the reviewers are now globally supportive, with some pending issues,

Reviewer 1 has a few remaining concerns, Specifically s/he is not satisfied with your reply concerning his/her question regarding that fact that the anti-DEC-205 conjugated OVA peptide does not activate OT-I cells, at variance with previous publication. Please discuss as requested.

The Reviewer also disagrees with your reply on his/her request to add CpG to help test the immunogenicity of the conjugated peptide. In this case should you have data in this respect, I would

strongly encourage you to include it in the manuscript. In alternative, please provide a reply and introduce appropriate cautionary statements in the manuscript.

Finally, Reviewer 1 maintains that OVA unconjugated peptide is an appropriate and non-redundant control. As above, should you have data, it would be best to include it in the manuscript, but I would be prepared to accept the introduction of appropriate cautionary statements in the manuscript.

Please also provide a full rebuttal to the Reviewer's comments as a separate file.

Considering the likely event that your manuscript will be accepted, I would suggest that you also deal with the following editorial requests at this stage:

1) We are now encouraging the publication of source data, particularly for electrophoretic gels and blots, with the aim of making primary data more accessible and transparent to the reader. Would you be willing to provide a PDF file per figure that contains the original, uncropped and unprocessed scans of all or at least the key gels used in the manuscript? The PDF files should be labeled with the appropriate figure/panel number, and should have molecular weight markers; further annotation may be useful but is not essential. The PDF files will be published online with the article as supplementary "Source Data" files. If you have any questions regarding this just contact me.

2) Every published paper now includes a 'Synopsis' to further enhance discoverability. Synopses are displayed on the journal webpage and are freely accessible to all readers. They include a short standfirst as well as 2-5 one-sentence bullet points that summarise the paper. Please provide the synopsis including the short list of bullet points that summarise the key NEW findings. The bullet points should be designed to be complementary to the abstract - i.e. not repeat the same text. We encourage inclusion of key acronyms and quantitative information. Please use the passive voice. Please attach this information in a separate file or send them by email, we will incorporate it accordingly.

3) Could you please provide a higher resolution version of panel E in Figure 2?

Please submit your revised manuscript within two weeks.

I look forward to seeing a revised form of your manuscript as soon as possible.

***** Reviewer's comments *****

Referee #1 (Remarks):

1) The author should perform immunization experiments in MyD88ko and TLR9ko mice with the DEC-205 targeting as well as the WT phages, to demonstrate that the increased immunogenicity of anti-DEC-205 phages is dependent on the TLR9-MyD88 pathway;

Author's response: These experiments have been performed and are reported in figure 3. They are described in results (page 8 of the revised text).

Referee comment: satisfied with the authors' response

2) The author should test the ability of scFv anti-human DEC-205 phages to activate the different human DC populations (in particular the CD141+ population and the pDC). This may of course require production of a new recombinant bacteriophage expressing a scFV recognizing the humanDEC-205, but it is critical to generate data in support of the proposed application of this technology for human use;
Author's response: This issue is of undoubted interest and as stated by the referee, would require the production of a new recombinant bacteriophage. We hope to address it in a future work.

Referee comment: satisfied with the authors' response

3) Figure 1B, C and D: it is unexpected that the anti-DEC-205 conjugated OVA peptide does not

activate OT-I cells as shown in a previous publication (Bonifaz et al., JEM 2004). How do the authors explain this result?

Author's response: Indeed a significant proliferative activity using NLDC:pOVA (anti-DEC205 mAb conjugated toOVA peptide) was observed in the adoptive transfer experiment (Figure 1B, p=0.039 vs control),while no IFN-γ production was observed (Figure 1 C). These results are in agreement with the
data previously reported by Bonifaz et al (JEM, 2004). With data reported in Figure 1, we would like to emphasize that, in an experimental setting characterized by the absence of exogenously added adjuvants, the proliferative activity induced by NLDC:pOVA is inferior to the proliferative activity induced by fd bacteriophage DEC-205-targeted particles displaying the OVA peptide.

Referee comment: This referee disagrees with the authors' interpretation of the results in Figure 1B. The proliferation of OT-I cells induced by anti-DEC-205 conjugated OVA peptide (corresponding to 1,6 ug of peptide) (around 15%) is not dramatic and the statistical significance should not be calculated compared to the PBS control but to an Isotype control antibody conjugated with OVA peptide or at least to the unconjugated OVA peptide, all controls missing in this experiment. This proliferation is basically identical to that induced by the LPS free Ovalbumin used as control and that is supposed to induce a limited proliferation compared to the processed peptide. In the cited reference (Bonifaz et al., JEM 2004) anti-DEC-205 conjugated to OVA protein (50 ng which is roughly 1/1500 molar equivalent of the OVA peptide used in the current manuscript) is able to induce proliferation in 100% of the adoptively transferred OT-I cells. This is a big difference compared to what the authors observe in their experimental setting that cannot be ignored since it could indicate that the anti-DEC-205 conjugated OVA peptide used in this manuscript is of poor quality and therefore not immunogenic. The author should discuss these discrepancies.

Addition of an adjuvant, such as CpG, could help to test the immunogenicity of the conjugated peptide.
Author's response: It has been previously described that addition of exogenous adjuvants as CpG increases the immunogenicity of antigens delivered by anti-DEC-205 mAb. In this manuscript we focused our attention on the use of the fd carrier as being able to confer a strong immunogenicity to the displayed antigenic determinants without the addition of exogenous adjuvant.

Referee comment: The referee understand that anti-DEC-205 conjugated OVA peptide is not the focus of this manuscript, but the addition of this control would help to understand if this reagent is immunogenic and can be considered an appropriate control in this experimental setting. Otherwise, addition of anti-DEC-205 conjugated OVA peptide cannot be used to interpret the results with the anti-DEC-205 phages.

Some controls are also missing in these experiments: immunization with the unconjugated peptide and with LPS free ovalbumin.
Author's response: We now report in Figure 1 the response to LPS free Ovalbumin (50 μg/ml), as a control of OT-I proliferative capability and specificity. In this context ,we reckoned that the use of unconjugated synthetic peptide as control was reduntant and we did not use it.

Referee comment: See comments above. OVA unconjugated peptide is the appropriate control which is therefore not redundant.

4) Figure 1 F and G: cytokine production induced by Poly I:C and CpG should be shown here
Author's response: In this figure we now report in all panels the use of LPS as a positive control. We consider redundant to report the use of Poly I:C as positive control. Concerning CpG, we report it as control in figure 3 (where response to MyD88-/- and TLR9-/- BMDCs in comparison to wild type BMDCs is illustrated).

Referee comment: The most appropriate positive control for IFN-a production by DC are either Poly I:C or CpG: it could help to compare the amount of this cytokine produced by anti-DEC-205 phages to that induced by TLR3 or TLR9 engagement in this experimental setting. However, LPS can be a surrogate positive control.

5) Figure 3: BMDC from WT mice need to be tested in parallel to KO cells in the same experiment;

Author's response: We have modified figure 3 according to Referee's suggestion.

Referee comment: satisfied with the authors' response

6) Figure 5C: image on the right (merge) seems identical to image in the middle (red fluorescence) not a merged image;
Author's response: This figure has been already amended in the previous submission.

Referee comment: satisfied with the authors' response

7) Page 4 lane 10: in the paper cited (Bonifaz et al., JEM 2004) immunogenicity of the anti-DEC205 conjugated OVA is increased by addition of anti-CD40, not by CpG or Poly I:C as indicated in the text.
Author's response: We agree with the Referee that we did only provided the reference concerning the use of anti-CD40. We now provide the missing references concerning the use of Poly I:C or CpG to induce the immunogenicity of antigen conjugated to anti-DEC-205 mAb.

Referee comment: satisfied with the authors' response

8) Page 4 lanes 22-23: "Phage administration has proven to be safe (Reardon, 2014)": this statement is misleading since no human test with this type of recombinant bacteriophages as a vaccine delivery system has been performed so far. The cited reference regards the use of phages to treat bacterial infections and is not appropriate here.
Author's response: The statement has been modified as follows:
Bacteriophages only infect and multiply with their specific host and currently the therapeutic use of bacteriophages is back on the agenda as bacterial resistance to antibiotics becomes widespread (Reardon, 2014). It should be mentioned that the use of the bacteriophage √X174 to assess specific antibody responses in patients with immunodeficiencies has been reported for many years, and is considered a safe, well-tolerated and clinically useful method (Smith et al, 2014). In theory, the administration of filamentous bacteriophage fd in humans should be also considered safe, even if no human tests with this type of recombinant bacteriophage as a delivery system have been performed so far.
Referee comment: satisfied with the authors' response

Referee #2 (Comments on Novelty/Model System):

The targeted bacteriophage antigen delivery system is innovative. In mice, the targetting appears effective at increasing immune responses. Until it is tested in humans, we do not know whether it will prove similarly immunogenic, and we do not know if it will be well tolerated and safe. Nevertheless, mouse experiments are a good early step.

Referee #2 (Remarks):

This revised manuscript adequately addresses the issues that I raised in my review of the original manuscript.

Referee #3 (Comments on Novelty/Model System):

The authors have addressed most of the issues raised by the reviewers, including mine. Therefore, I suggest that this paper should be published.

2nd Revision - authors' response 19 March 2015

Authors' Point-by-Point Reply to Reviewer Criticisms:

Referee #1

1) Addition of an adjuvant, such as CpG, could help to test the immunogenicity of the conjugated peptide.
Author's response: It has been previously described that addition of exogenous adjuvants as CpG increases the immunogenicity of antigens delivered by anti-DEC-205 mAb. In this manuscript we focused our attention on the use of the fd carrier as being able to confer a strong immunogenicity to the displayed antigenic determinants without the addition of exogenous adjuvant.

Referee comment: The referee understand that anti-DEC-205 conjugated OVA peptide is not the focus of this manuscript, but the addition of this control would help to understand if this reagent is immunogenic and can be considered an appropriate control in this experimental setting. Otherwise, addition of anti-DEC-205 conjugated OVA peptide cannot be used to interpret the results with the anti-DEC-205 phages.

Taking into accounts the referee suggestion's we have inserted in fig 1 and in results (page 5) data obtained with anti-DEC-205 antibody conjugated to OVA$_{257-264}$ peptide administered in the presence of CpG.
The text was thus amended as following, discussing these results in comparison to previous reported data:

"The proliferative activity induced by the OVA$_{257-264}$ antigenic determinant delivered by anti-DEC-205 is low in comparison to a previous report describing the proliferation of OT-I T cells stimulated by an anti-DEC-205 antibody carrying Ovalbumin protein (Bonifaz et al, 2004); while in agreement with this report (which showed lack of IFN-g production using Ovalbumin conjugated to an anti-DEC-205 antibody) we did not observe production of IFN-g by OT-I T cells stimulated with anti-DEC antibody delivering the OVA$_{257-264}$ peptide (NLDC:pOVA, ***Fig 1, panels B,C****). When NLDC:pOVA conjugate was administered in the presence of the adjuvant CpG, a higher proliferative response as well as IFN-g production by OT-I T cells were observed (****Fig 1, panels B,C****). These data indicate that, in an experimental setting characterized by the absence of exogenously added adjuvants, the fd bacteriophage DEC-205-targeted particles confer strong immunogenicity to the displayed antigenic determinant."*

2) Some controls are also missing in these experiments: immunization with the unconjugated peptide and with LPS free ovalbumin.
Author's response: We now report in Figure 1 the response to LPS free Ovalbumin (50 μg/ml), as a control of OT-I proliferative capability and specificity. In this context ,we reckoned that the use of unconjugated synthetic peptide as control was reduntant and we did not use it.

Referee comment: See comments above. OVA unconjugated peptide is the appropriate control which is therefore not redundant.

We agree with the referee that unconjugated OVA peptide may represent an appropriate control. However, concerning the aim of our experiment, we reckon that the controls used here (NLDC:OVA plus CpG, LPS free ovalbumin and vehicle (PBS)) are sufficient to prove that the OVA peptide delivered by bacteriophage fd in the absence of exogenous adjuvants is highly immunogenic, being also able to induce IFN-g production.
